# Atmospheric Sampling Mass Spectrometers Activate and Ionize Neutral Water Microdroplets to MeV Energies and up to 200,000+ Charges: Implications for Water Stability and Unusual Chemistry in Microdroplets

**DOI:** 10.1021/acscentsci.5c01518

**Published:** 2025-10-31

**Authors:** Matthew S. McPartlan, Casey J. Chen, Conner C. Harper, Zachary M. Miller, Julian Robles, Veena S. Avadhani, Randall E. Pedder, Luke J. Metzler, Evan R. Williams

**Affiliations:** † Department of Chemistry, University of California, Berkeley, California 94720-1460, United States; ‡ Ardara Technologies LP, Ardara, Pennsylvania 15615, United States

## Abstract

Neutral water microdroplets formed by condensation were introduced into a mass spectrometer with a standard stainless-steel capillary atmospheric interface. Placing volatile samples near the mass spectrometer inlet led to spectra comparable to those of electrospray ionization of the same compounds. Neutral microdroplets introduced into a charge detection instrument through a nearly identical atmospheric sampling interface resulted in charged microdroplets with diameters between 165 nm (∼3,000 charge detection threshold) and 2.8 μm (200,000+ charges). The vast majority of 100,000+ aqueous microdroplets analyzed were positively charged. Aerodynamic acceleration led to average velocities of ∼270 m/s and ∼210 m/s for the smallest and largest microdroplets, respectively, with corresponding energies ranging from a few MeV to 300+ MeV for the largest microdroplets. Current measured on the capillary and other results indicate that interactions between the neutral microdroplets and the capillary can strip off hundreds of thousands of electrons to form positively charged droplets. Negatively charged microdroplets indicate a minor process in which neutral microdroplets are broken up into smaller microdroplets of both polarities. The MeV+ energy involved in these interactions is sufficient to drive many chemical reactions and may lead to unusual and unexpected chemistry when analysis is done using mass spectrometers with atmospheric sampling interfaces.

## Graphical abstract

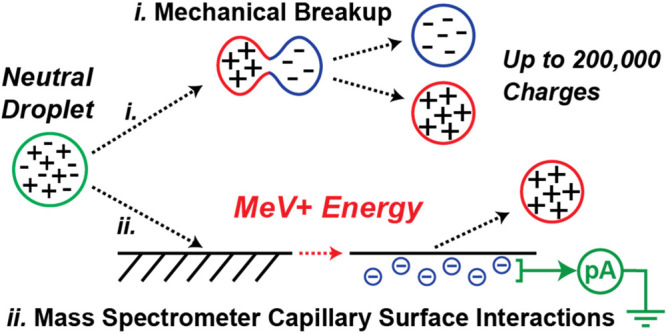

## Introduction

Although many different techniques have been used to investigate accelerated reactions and unusual chemistry in microdroplets, a significant fraction of studies in which evidence for unusual droplet chemistry has been reported involve mass spectrometry detection.^1–11^ Analysis via mass spectrometry requires gas-phase ions that are typically produced using ionization sources that provide some form of activation. For example, an electron is removed from a molecule using energetic electrons in electron ionization and energetic electrons or photons are often used to initiate chemical ionization. Pulsed lasers are used in photoionization or desorption/ionization sources, e.g., matrix assisted laser desorption/ionization. In pneumatic nebulization, which was used in many investigations into reaction acceleration and unusual chemistry,^9–12^ compressed gas disperses liquid water into small microdroplets that are both positively and negatively charged.^13,14^ Both increasing the surface area of liquid water by forming microdroplets and separating microdroplets with opposite polarity requires substantial energy that is provided by expansion of the compressed gas. Electrospray ionization (ESI) produces highly charged droplets and has also been used in many studies in which unusual chemistry in microdroplets has been reported.^5,8,12,15^ The energy that is required to charge and disperse liquid water into small microdroplets is provided by the ESI potential and current. Energy is also added to microdroplets through the collisional activation or thermal heating that occurs in atmospheric sampling mass spectrometer interfaces. This leads to solvent evaporation and ultimately produces bare gaseous ions for mass analysis.

Thus, a recent report by Zhang and co-workers^6^ showing that neutral water droplets formed by adding dry ice into water at ambient pressure can lead to spontaneous ion formation and unusual chemistry initially seems surprising. The authors used a mass spectrometer with a standard atmospheric pressure ion introduction system and proposed that ions are spontaneously formed from neutral droplets due to the high intrinsic electric field at the droplet surface. Moreover, they state that these neutral droplets are not activated and show that the same reactions and reaction acceleration that has been reported to occur in charged droplets also occurs in these neutral droplets. For example, they report a *m*/*z* 36 ion, that they attribute to (HO·-H_3_O)^+^, is formed in high abundance from pure water microdroplets that are uncharged, and an ion that is +17 Da higher in mass than protonated melatonin that they attribute to a reaction with OH·, is formed from neutral aqueous droplets containing melatonin.^6^ These ions have been proposed as evidence for spontaneous production of abundant hydroxyl radicals due to the high intrinsic electric field at the surface of water droplets.^6,9,12,16^ Although (HO·-H_3_O)^+^ can be formed in electrical discharges or plasmas,^7,17^ accurate mass measurements show that the *m*/*z* 36 ion produced from charged aqueous nanodrops and microdroplets that are not intentionally activated is NH_4_^+^(H_2_O) and not (HO·-H_3_O)^+^ in any structural form.^18,19^ Accurate mass and collision induced dissociation experiments indicate that the +17 Da addition to protonated melatonin and caffeine is due to ammonia adduction and not a reaction with hydroxyl radicals.^20^ Mass spectrometers are sensitive instruments, and the source of trace ammonia in these experiments can be from human breath,^21–24^ dermal emission,^21,25^ pickup of ammonium salts commonly used in separations and native mass spectrometry, or solution contamination.^20^

An important question to answer in understanding chemistry thought to occur in neutral water droplets is how are ions formed spontaneously from neutral droplets in experiments that use mass spectrometry detection? Does the intrinsic electric field at the surface of unactivated neutral aqueous microdroplets cause spontaneous ionization as proposed? ^6,9^ Or are there alternative mechanisms for charging in atmospheric sampling mass spectrometer interfaces that provide energy, which can readily explain these and many prior observations^26–28^ in the analysis of uncharged liquids with mass spectrometry?

Here, we show that highly charged aqueous nanodrops and microdroplets are formed from neutral droplets in atmospheric sampling interfaces of mass spectrometers. A custom designed charge detection instrument capable of measuring large (up to micron-sized) charged droplets was used to identify the extent of activation and ionization that occurs with these neutral droplets. These measurements show that there are two different processes that can lead to the formation of highly charged aqueous microdroplets with up to 200,000+ charges from initially neutral microdroplets. These results indicate that prior results purporting to show reactions in unactivated neutral water microdroplets almost certainly occur in highly charged droplets formed by activation of neutral droplets in the atmospheric pressure interfaces of mass spectrometers. The extent of droplet charging with these two mechanisms is similar to that of droplets produced by electrospray ionization, a technique known to produce aqueous droplets with an extent of charging close to and even above the Rayleigh limit.^29–32^

## Results and Discussion

### Charge Detection with an Array Detector

Atmospheric sampling mass spectrometers are widely used to characterize unsolvated gaseous ions formed from droplets. Information about solution composition and chemistry can be inferred from these data. However, the effects of the atmospheric sampling interface on these measurements have not been fully characterized. To investigate how unsolvated gaseous ions can be produced from neutral droplets, a custom designed and constructed charge detection instrument was used to directly measure what occurs to neutral droplets as they transition from ambient pressure into a mass spectrometer with an atmospheric sampling interface. This instrument has a single-pass array detector consisting of 24 conductive tubes that are used to detect charged particles, such as nano or microdroplets, as they pass through the array (Figure 1a). This instrument is similar to other linear array charge detection mass spectrometers that can measure the masses of ions in the hundreds of megadalton to teradalton range.^33–37^ *All ion optics were grounded in these experiments so that there were no electrostatic effects on charged droplets passing through this device.* The atmospheric interface is similar to those used in commercial instruments for experiments in which unusual water microdroplet chemistry has been reported.

**Figure 1:**
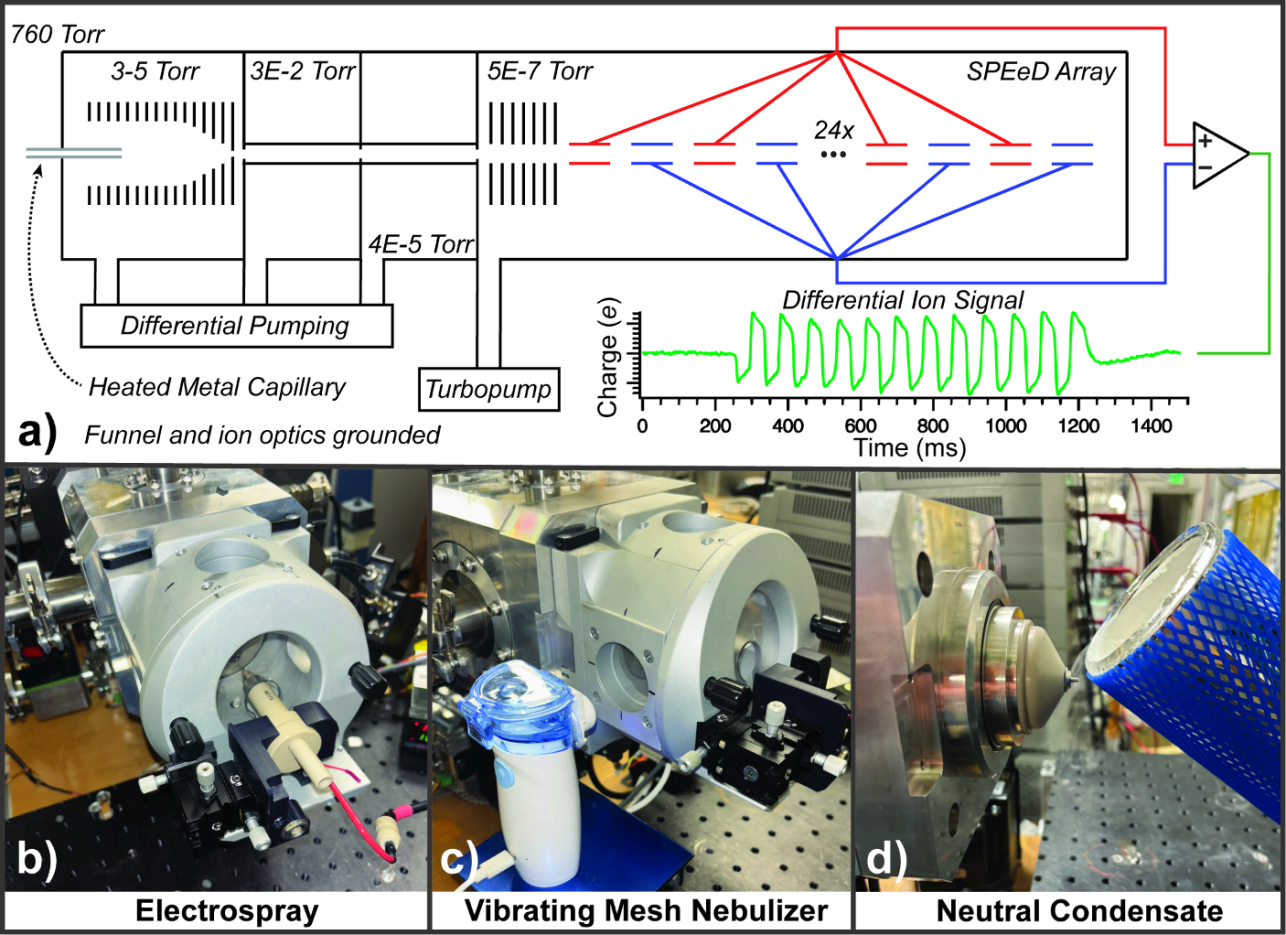
Charge detection instrument showing (a) a schematic diagram of the charge detection instrument that has a differential array detector with 24 detector elements and the resulting signal from an ion passing through the array (in green); photographs of various droplet formation methods used including (b) electrospray ionization, (c) bipolar droplets generated by a mechanical vibrating mesh nebulizer, and (d) neutral droplets formed as condensate from a hand-held dewar of liquid nitrogen.

To illustrate the information that can be obtained using this method, an example of charged droplets formed via positive and negative electrospray ionization (ESI) of an aqueous 200 μM NaCl solution is shown in Figure 2a. The addition of NaCl substantially increases solution conductivity, making ESI droplet formation more consistent. Charged droplets that pass through the array detector induce a signal within each detector tube in the array. The differential measurement between alternating detector tubes produces a signal resembling a square wave. The decay in the induced square wave is a result of RC filtering within the detector and amplifier circuitry. The droplet polarity is determined from the initial pulse of this signal, i.e., the signal phase. Positively charged droplets produce a positive initial pulse (Figure 2a, red data) whereas a negative initial pulse is induced for negatively charged droplets (Figure 2a, blue data).

**Figure 2:**
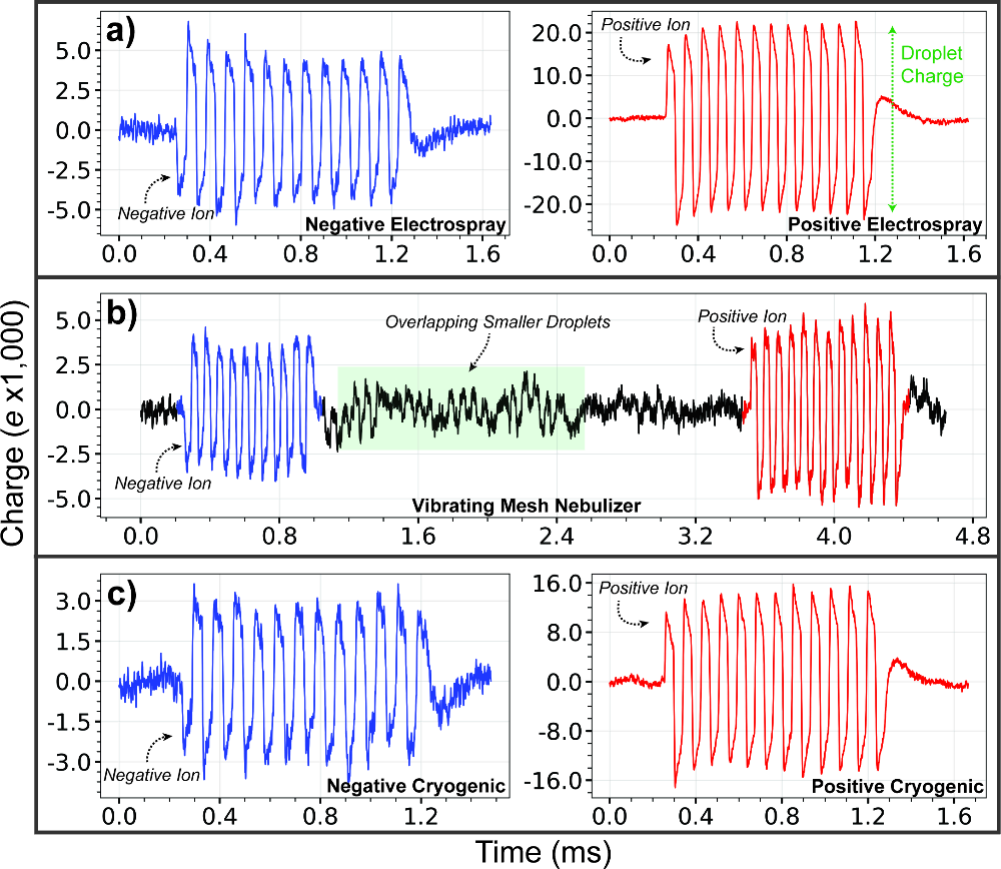
Signals generated by charged droplets that pass through the array detector of the charge detection instrument with droplets generated by (a) negative (blue data) and positive (red) electrospray, (b) negative (blue data) and positive (red data) droplets generated by a vibrating mesh nebulizer and measured consecutively within ∼4 ms using identical instrument conditions (signal in green shaded box is from overlapping signals of multiple small droplets that passed through the detector array at overlapping times), and (c) negative (blue data) and positive (red data) droplets formed from initially neutral droplets produced by condensation with liquid nitrogen. Polarity of the droplet is determined from the initial phase of the signal.

The ion charge can be obtained from the signal amplitude (Figure 2a, green arrow). A charge calibration was done with an accurate externally generated test signal (Supporting Information). The accuracy of this calibration was confirmed by forming ions from 300 nm polystyrene nanospheres using ESI with the charge detection instrument operated as a mass spectrometer. The average charge of the polystyrene nanospheres was ∼5,100 *e*, which is consistent with highly accurate charge measurements made using an electrostatic linear ion trap charge detection mass spectrometer.^38^ The amplitude of the signal induced by the positively charged droplet in Figure 2a (red data) indicates that this droplet had a charge of ∼43,600 *e*.

The microdroplet size can be estimated from the measured charge. Rayleigh predicted that the maximum charge (*z*_*R*_)^29^ that a spherical droplet of radius *R* and surface tension γ can sustain before fission becomes likely (*ε*_0_ is the permittivity of free space) is given by eq 1:

1 $$z_{R}={8\pi \left(\varepsilon_{0}\gamma R^{3}\right)}^{1/2}$$

Positively charged water droplets produced by ESI and introduced into a charge detection mass spectrometer have charges ranging between 0.4 and 1.6 times the Rayleigh limit charge, with an average charge slightly above the Rayleigh limit.^32^ This indicates that a lower limit to the droplet size can be determined by using the measured charge in place of *z*_*R*_ in eq 1. If the positive ESI generated water droplet (Figure 2a) is charged at the Rayleigh limit (*z*_*R*_ = 43,600 *e*), it would have a diameter of ∼1 μm and a mass of ∼302 GDa. It is important to note that the droplet size determined by this method is a lower limit. If these droplets are charged substantially below *z*_*R*_, the mass (and hence diameter) of the droplets would be even larger than we report.

The velocity of each droplet is obtained from the transit time between detector tubes, i.e., the time between pulses (Supporting Information). The velocity of the positively charged ∼302 GDa droplet was 265 m/s. The kinetic energy of this ion, determined from its approximate mass and measured velocity, was ∼110 MeV. The negatively charged droplet shown in Figure 2a (blue data) had a similar velocity (264 m/s) but had significantly less charge (9,810 *e*), indicating that this is a much smaller droplet (∼365 nm with a mass of ∼15 GDa). This mass and velocity correspond to a kinetic energy of ∼5.5 MeV. These energies are also lower limits to the true value. Droplet charging substantially below *z*_*R*_ would mean that these droplets have even higher kinetic energies owing to the underestimation of droplet mass.

What is the origin of this seemingly high kinetic energy? The atmospheric interface generates an expansion whereby atmospheric air molecules move at similar velocities through the steel capillary.^35,39^ Large microdroplets move along with the air, albeit at slower velocities. The slip velocity, the velocity difference between a particle’s velocity and that of the surrounding gas flow, depends on particle size.^39,40^ Thus, the source of energy is the pressure differential across the steel capillary that is maintained using a mechanical vacuum pump. While this kinetic energy appears high, for the positively charged droplet with ∼110 MeV of energy shown in Figure 2a (red data), this corresponds to just ∼0.007 eV per water molecule.

To establish that both positively and negatively charged droplets can be measured in these experiments with no change in instrument settings (there are no electrode voltages used in these experiments), a vibrating mesh nebulizer that produces droplets with initial diameters between 2 and 20 μm was positioned 10 cm from the source inlet (Figure 1c). Mechanical breakup of water is well-known to produce both positively and negatively charged droplets^42^ and gaseous ions of both polarities can be formed from molecules contained within these respectively charged droplets. Figure 2b shows a negatively charged droplet passing through the detector array followed by a positively charged droplet ∼2.5 ms later. The charges on these droplets are −7,560 *e* and +9,950 *e*, respectively, corresponding to droplet diameters of ∼306 nm and ∼368 nm, respectively (eq 1). These results demonstrate the ability to detect charged droplets of either polarity consecutively with no change in experimental parameters. Also shown between ∼1.3 and 2.3 ms are overlapping signals of some smaller droplets as they pass through the detector array at the same time (Figure 2b, green box).

### Cryogenic Neutral Microdroplet Ionization

Microdroplets were formed by condensation of molecules in ambient air above an open liquid nitrogen dewar (Figure 1d). Condensation and microdroplet formation occur due to the cold temperature of the N_2_ gas. The droplet composition is primarily water due to its abundance in ambient air, but other ambient molecules can condense or otherwise incorporate into these droplets. The condensate microdroplets were introduced into the mass spectrometer by placing a dewar of liquid nitrogen ∼1–2 cm from the inlet of the charge detection instrument (Figure 1d, Video SV1). Both positively and negatively charged microdroplets were detected. Examples of signals from a positively charged (28,800 *e*) and a negatively charged (6,280 *e*) droplet produced with cryogenic condensation are shown in Figure 2c (red and blue data, respectively). These signals were acquired during the same experiment under identical conditions.

The charges and velocities for a total of 104,443 noninterfering cryogenically generated microdroplets were measured. A high charge threshold (∼3,000 *e*, corresponding to a microdroplet diameter of 165 nm) was used to process these data with automated software (details in Supporting Information) to make microdroplet characterization more robust and to more effectively eliminate overlapping signals so that only noninterfering signals corresponding to individual microdroplet ions were considered. The vast majority of these microdroplets were identified as positively charged (101,855 or 97.5%) by our automated software analysis, and 2,588 (2.5%) were initially identified as negatively charged. However, the automated software misidentified the polarity of a small fraction of these droplets.

To learn more about the misidentification rate, 2,000 signals for microdroplets that the automated software identified as positively charged were manually reviewed. This analysis revealed that 1,993 microdroplets were correctly identified as positive, 5 were ambiguous, and 2 were negative microdroplets misassigned by the automated software. This implies an error rate of 0.35%, but this underestimates the true error rate. A much higher error rate was obtained for the droplets that the software identified as being negatively charged. The signals of all 2,588 microdroplets that our automated software identified as negatively charged were manually inspected. 1,757 of these signals were from positively charged microdroplets that were misidentified by the automated software. A total of 493 were correctly identified as negatively charged microdroplets and the identities of the remaining 338 ions were ambiguous. The primary origin of this high misidentification rate for negatively charged droplets is overlapping ion signals. When a small ion enters the detector array prior to a larger ion and the signals overlap, the baseline can be adversely affected which can lead to incorrect initial pulse identification by the automated software. This effect can often be identified when manually inspected, as was the case here for 1,757 droplets but not for the 338 ambiguous droplets. This problem of misidentification is much more significant for negatively charged ions because they are less highly charged, making the effects of interference more difficult to distinguish. This is consistent with results from our manual inspection of the positively charged ions where the average charge is significantly higher and the error rate is low. There is likely a similar error rate for the lowest charged positive ions. Thus, while the true error rate is difficult to ascertain, the vast majority of microdroplets are positively charged.

The distribution of charge for droplets of each polarity produced by cryogenic microdroplet ionization is shown in Figure 3. For the positively charged droplets, the distribution is peaked at ∼7,000 charges and extends out to over +200,000 *e*, after which the digitizer was saturated. This indicates that even more highly charged droplets were produced but their exact charge could not be measured. The origin of the peak is likely due to the charge cutoff (∼3,000 *e*) that was used in our automated analysis to reduce misidentification of the droplet charge polarity and magnitude. Thus, droplets with fewer charges were also formed in these experiments (e.g., the droplets highlighted by the green box in Figure 2b). The negatively charged droplets were less highly charged with a peak around 7,000 *e* but tail off much more rapidly, even after considering the fewer number of negatively charged droplets.

**Figure 3:**
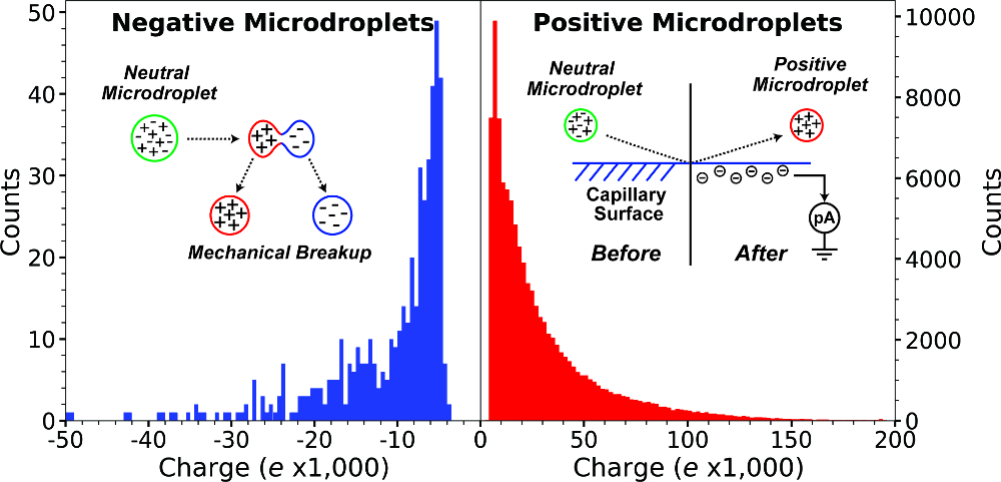
Population of droplets originating from neutral droplets generated by condensation with liquid nitrogen as a function of individual droplet charge. Negatively and positively charged droplet data are shown in blue and red, respectively. Inset on right illustrates the mechanism for how the vast majority of droplets that are positively charged are formed. Inset on the left illustrates how the significantly less common negatively charged droplets (and some positively charged droplets) are formed. The negative droplet data includes only signals that were manually verified. The positive droplet data includes all droplets that were automatically identified by the analysis software.

The droplet velocity as a function of mass is shown in Figure 4a. There is significant variability in the velocities at a given mass. However, there is a clear relationship between droplet velocity and droplet mass. The average velocity as a function of mass (Figure 4a, red line) rapidly decreases from ∼270 m/s for the smallest droplets and approaches a velocity of ∼210 m/s for the largest droplets. This is consistent with larger particles having higher slip velocities^39,41^ and with the slightly higher velocities (400 m/s) reported by Dugourd and co-workers using a similar instrument for smaller analytes (<50 MDa).^36^ A distribution of the number of droplets as a function of mass is shown in Figure 4b (green line). This shows that most droplets are smaller than 200 GDa (<860 nm diameter) but there are some droplets with diameters beyond 2.8 μm. From these data, the kinetic energies of these ions as a function of mass were determined (Figure 4b, purple line). The kinetic energies of droplets increase nearly linearly with mass (except at very small sizes, Figure S1) and range from a few MeV to 300+ MeV.

**Figure 4:**
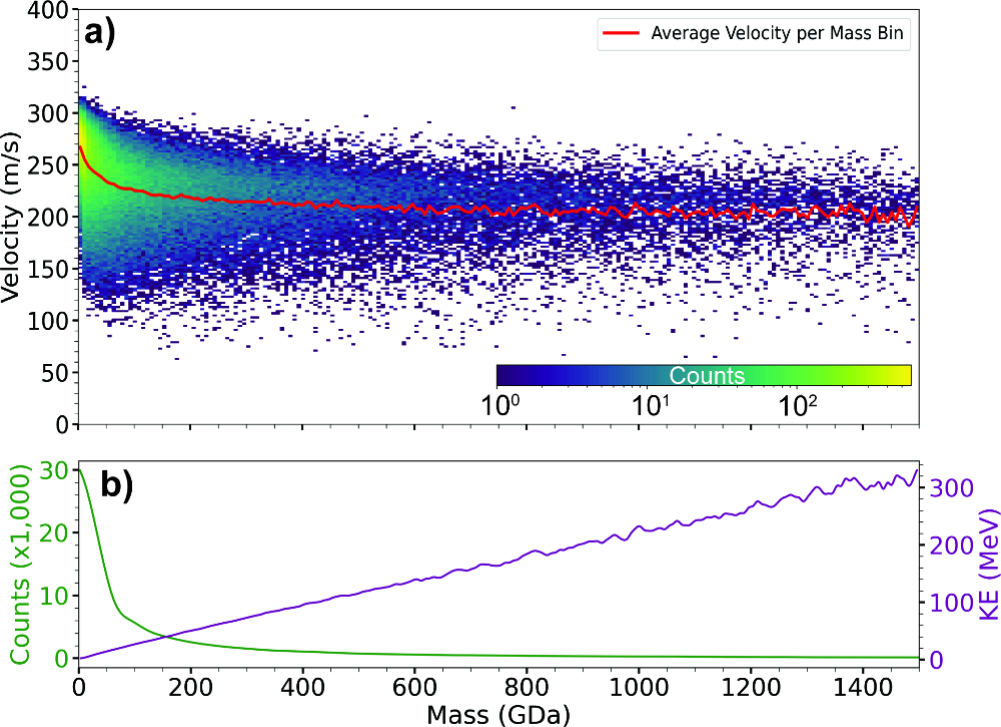
Data for positively and negatively charged droplets formed by condensation using liquid nitrogen showing (a) velocity as a function of droplet mass, indicating a wide range of velocities for droplets with a given mass but a clear trend in average velocity (red line) as a function of droplet mass, and (b) distribution of droplets as a function of mass (green line) and kinetic energy of droplets determined from the average velocity and estimated mass of the droplets (purple line).

### Origin of Neutral Droplet Charging in Mass Spectrometry

We set out to identify where the charging of these initially neutral droplets occurred. A negative current between 10 pA and 1.1 nA was measured on the metal capillary of the atmospheric interface, indicating that a substantial number of electrons or anions are removed from the droplets in this region. No current was measured without the liquid N_2_. These results indicate that the initially neutral droplets are the source of the measured current. We attribute the large variability of the current measurements primarily to movement of laboratory air that can reduce the number of droplets entering the instrument.

To support our hypothesis that the droplets formed by condensation of water were initially uncharged, a copper mesh was placed across the top end of a vertical 2″ diameter PVC tube. At the bottom of this tube, a 90° bend directed droplets toward the mass spectrometer inlet ∼5 cm away. Droplets were introduced at the top of the tube with a voltage of ±5.0 kV or ground (0 V) applied to the copper mesh and the current on the metal capillary was measured for 5 s (Figure S2). There was no discernible difference in either the current or the number of ions passing through the detector array, indicating that the droplets formed by condensation are not initially charged. Similar results were obtained when a copper plate with a 0.5 cm diameter hole was placed in front of the atmospheric interface. Application of ±4.5 kV to this plate had no measurable effect on the number of ions passing through the array detector. These results provide additional support that the droplets formed by condensation were initially neutral prior to entering the charge detection instrument and that the PVC tube did not lead to charging of these droplets.

Contact electrification of water is well-known and can occur from ion or electron transfer between a liquid and a solid.^43,44^ To strip electrons (or anions) from neutral droplets inside the metal capillary, interactions between these droplets and the capillary surface must occur. Separate experiments were performed using a linear electrostatic ion trap charge detection mass spectrometer^38,45^ to confirm that droplet-capillary interactions do occur even with initially charged droplets. In these experiments, a small volume of a dilute polystyrene 50 nm diameter nanosphere sample was deposited inside the capillary and dried. Pure water nanodrops formed by ESI were subsequently introduced into the instrument. Signal for polystyrene nanospheres was observed. These ions can be unambiguously identified as polystyrene nanospheres based on their mass and frequencies of ion motion that do not change significantly with time, indicating that these ions are not hydrated to a measurable extent (Figure S3). In desorption electrospray ionization (DESI), solvent droplets interact with surfaces where they can pick up analyte molecules for introduction into a mass spectrometer.^46^ DESI has been reported to occur for small molecules at the walls of transfer tubes in mass spectrometers.^47^ In our charge detection mass spectrometry experiments, the charged water droplets must interact with the surface of the stainless-steel capillary of the mass spectrometer interface to pick up the previously deposited polystyrene nanospheres. These droplet-capillary interactions almost certainly occur from glancing collisions and can lead to the pickup of analyte molecules as well as the removal of tens to hundreds of thousands of electrons from neutral water droplets that interact with the metal capillary. The energy for this process is provided by the high kinetic energy of the droplets. Even though the energy per water molecule is very small and insufficient to cause ionization of a single water molecule, the MeV energy of the ensemble of water molecules making up a large droplet is sufficient to drive high energy reactions.

To determine whether water or other small molecules that might be condensed on the capillary at 30 °C are affecting these results, the neutral droplet experiments were repeated with the capillary at 140 °C. Although the temperature of the capillary surface is lower due to cooling by ambient air, this temperature is sufficient to form desolvated ions in our CDMS instrument that has an identical interface.^38,48^ The charge and velocity of 133,790 droplets were characterized by the automated software without manual validation. Under these conditions, the vast majority of ions were positively charged and had a similar charge distribution (Figure S4) as the data obtained at 30 °C. These data indicate that contact electrification occurs through direct interactions between the water droplets and the surface of the steel capillary.

In contrast to the positive ions that are formed by removal of electrons from the neutral water droplets, the rarity of the negatively charged droplets suggests that they may be formed by a different mechanism, although we cannot rule out contact electrification. When droplets pass through the metal capillary interface, the lower pressure combined with shear forces from molecular flow could lead to breakup of uncharged droplets. This is analogous to the mechanical breakup of liquid water using a variety of methods that lead to formation of both positive and negative droplets. A similar mechanism has been proposed to occur in heated metal interface capillaries in droplet assisted ionization.^49–51^ In these and similar experiments, droplets containing analytes are produced by breakup of water with^50,52,53^ or without^49,51^ additional inductive ionization. The mechanical breakup of neutral water microdroplets appears to be a minor mechanism for charging in the mass spectrometer interface, but one that can generate both negatively and positively charged droplets.^54,55^ The small number of negatively charged droplets and the lower charge of these droplets (Figure 3) may also be due in part to stripping of electrons from these negatively charged droplets and even charge reversal may occur to produce positively charged droplets.

Our results are consistent with the pioneering work of Trimpin, McEwen, and co-workers in which they showed that introduction of solvent containing analytes, including large proteins, directly into a heated capillary of a standard mass spectrometer atmospheric interface resulted in multiple charging of analytes similar to that produced by ESI.^26–28^ With an unheated or heated capillary, a positive current on a downstream electrode was measured, indicating that net positive ions or droplets were generated in the capillary.^56^

### Cryogenic Ionization Mass Spectrometry

To confirm that gas-phase analyte ions can be produced from neutral droplets introduced into a mass spectrometer with an ambient sampling source, a sample of 1,12-dodecanediamine was placed ∼2 cm below the inlet capillary of a mass spectrometer. This compound was chosen because it has a nonzero vapor pressure (bp = 304 °C)^57^ and both singly and doubly protonated molecules can be produced by ESI. Mass spectral data were acquired for over 1 min in the absence of liquid N_2_ and no ions were observed (Figure S5a). This demonstrates that this compound does not spontaneously ionize to a measurable extent. A dewar of liquid N_2_ was placed near the mass spectrometer inlet and condensate droplets were formed near the entrance of the mass spectrometer (Figure S6; Video SV2). The resulting mass spectrum is shown in Figure 5a. The most abundant ions are the protonated (*m*/*z* 201) and the doubly protonated (*m*/*z* 101) molecule. A singly charged ion at *m*/*z* 130 is also observed. This ion was formed prior to introduction of 1,12-dodecanediamine near the inlet with condensate from liquid N_2_ (Figure S7) and this ion was not formed by ESI of a solution of 1,12-dodecanediamine (Figure S8). Both results indicate that the *m*/*z* 130 ion is a contaminant in the laboratory air. Assuming that the molecule is protonated, the mass indicates that the compound has one or more nitrogen atoms, suggesting that this molecule likely has a high gas-phase basicity and can be readily ionized.

**Figure 5:**
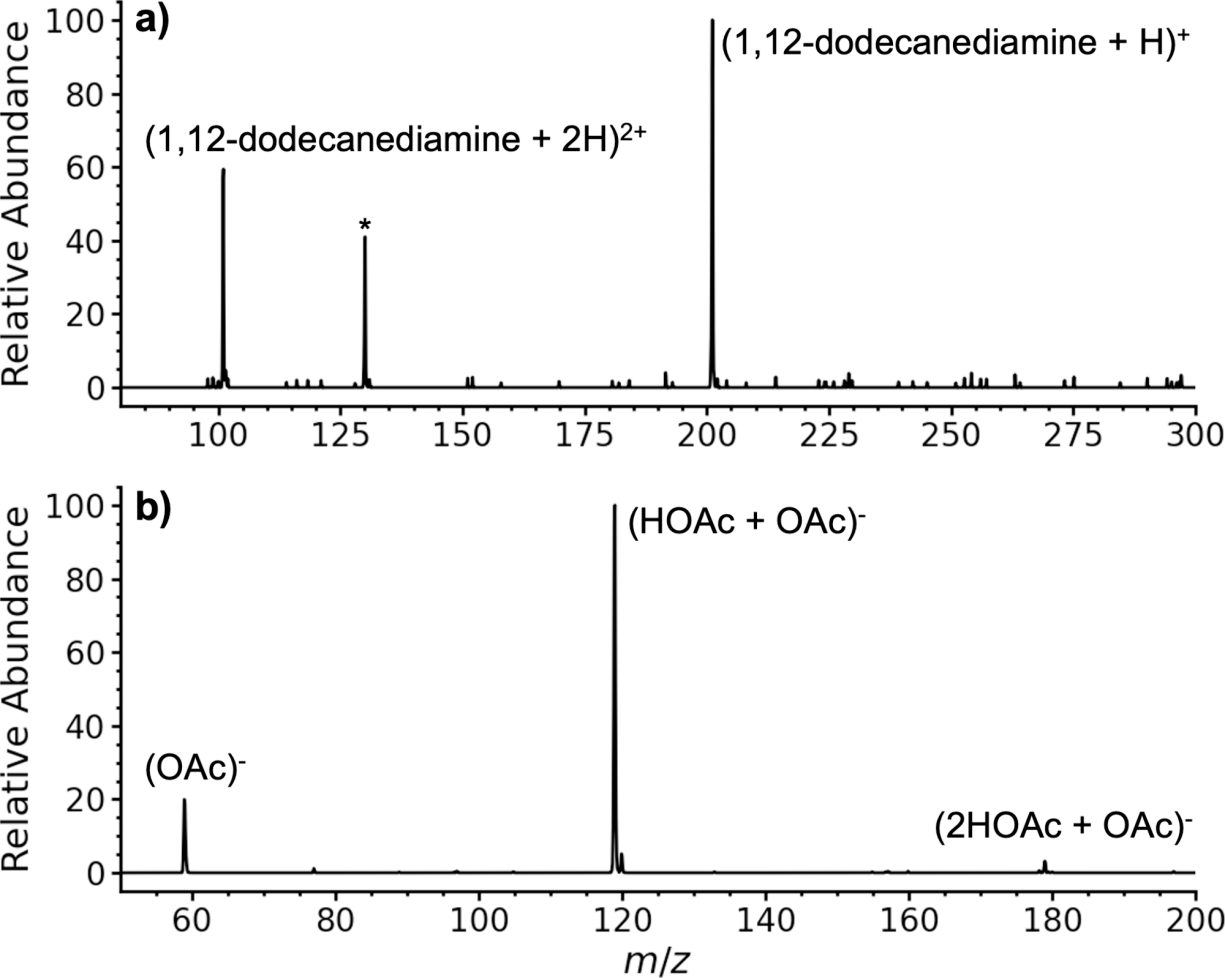
Cryogenic ionization mass spectra of vapor from (a) 1,12-dodecanediamine and (b) acetic acid with neutral droplets formed from condensation with liquid N_2_. Asterisk at *m*/*z* 130 in (a) corresponds to a background ion originating from laboratory air (see text). Spectra were obtained using the Velos Pro dual pressure linear ion trap of an Orbitrap Elite instrument (Supporting Information).

The appearance of doubly protonated 1,12-dodecanediamine in the cryogenic ionization mass spectrum indicates that some of the vapor from this compound is incorporated into the droplets and that the droplets from which these gaseous ions were formed must have had more than a single charge. This is consistent with the highly charged droplets observed in the cryogenic droplet charge detection experiments. The lower abundance of the doubly charged ion in the cryogenic ionization mass spectrum (Figure 5a) than in the ESI mass spectrum (Figure S8) indicates that some of the singly protonated ions in cryogenic ionization are also formed by proton transfer from protonated water, water clusters, or the ionized droplet itself. Increasing the temperature of the interface capillary from 40 to 100 °C led to a similar spectrum and an approximately 3-fold increase in the signal (Figure S9). This increased signal is consistent with improved desolvation at higher temperature leading to more efficient gaseous ion formation.

Similar experiments were performed with acetic acid that was added to a Kimwipe and placed near the inlet of the mass spectrometer. No ions were observed in the mass spectrum without liquid N_2_ for over one min (Figure S5b), but abundant acetate and complexes of acetate with acetic acid were formed with condensate from liquid N_2_ (Figure 5b). The signal intensity is much lower than for the positive ion data, but the observation of anions confirms that the negatively charged cryogenic ionization droplets measured using the charge detection instrument can lead to negatively charged gaseous ions.

### Are Ions Spontaneously Formed from Unactivated Water Microdroplets?

It is possible that not all neutral droplets become highly charged via our two proposed mechanisms. Neutral droplets or droplets with just a single charge cannot be measured with the charge detection array. We set out to address the question of whether there are other mechanisms whereby a single or doubly charged positive ion can be spontaneously produced from an unactivated neutral water droplet. To spontaneously produce a positively charged gaseous ion from a neutral droplet, a charge separation process must occur. The vertical and adiabatic ionization energies of liquid water are ∼11.2–11.7 and 10.1 eV,^58,59^ respectively, so spontaneous ionization by removal of an electron from a large neutral droplet should not occur. To confirm that no stray high-energy photons caused measurable ionization of the neutral water droplets in our experiments, an enclosure that blocked light from reaching the droplets was constructed. No effect on the formation of gaseous 1,12-dodecanediame ions from neutral condensate was observed, indicating that light is not responsible for ionization in these experiments (Figure S10).

It has been proposed that the intrinsic electric field at the surface of a water droplet whether charged or uncharged, can cause an electron to spontaneously detach from OH^–^ (or H_2_O) to form a solvated electron and a positive ion or radical.^8,10,60–62^ Formation of a gaseous positively charged ion requires separation from a negatively charged droplet if produced by ion emission from the droplet, or the odd electron ion would need to separate from an electron if the ion is formed by evaporation of solvent. Either process requires substantial energy and should not spontaneously occur to a significant extent in an unactivated droplet. Ion emission from an uncharged droplet must overcome both the ion solvation energy and the Coulombic attraction between a positive ion and a negatively charged droplet. This process differs from the well-known technique of field ionization, where the voltage on an electrode is regulated through applied current. Positively charged ions formed by electron tunneling are repelled from the electrode. In contrast, spontaneous ionization of a droplet that leads to a positive ion and negatively charged droplet must overcome the long-range Coulombic attraction between oppositely charged particles, a process that requires energy to be deposited into the system.

Microdroplets have different properties than bulk liquid water, and much of the unusual chemistry reported in microdroplets has been attributed to the high intrinsic electric fields at the surface.^6,8–10^ Infrared photodissociation spectra of small ion-containing aqueous nanodrops indicate that the electric field at the surface of a microdroplet should be essentially the same as that at the bulk air–water interface.^63^ The spectral signature of some water molecules that have a free–OH stretch at the surface of ion-containing nanodrops shows that this frequency depends on both the orientation of the surface water molecules and on a Stark shift due to the electric field at the nanodrop surface.^63–65^ The electric field is due to both the ion and the electric field induced by the oriented water molecules at the droplet surface. For nanodrops that contain +1, +2, −1, or −2 ions, the charge on the ion can induce a water orientation effect at the droplet surface, but this effect becomes negligible for clusters with more than a few hundred water molecules.^63^ Above this size, the change in free–OH frequency as a function of cluster size is consistent with a Stark shift due to the electric field of the ion and the electric field induced by water orientation at the surface that both depend on cluster size. Extrapolation of these data to infinite droplet size indicates a free–OH stretch frequency of 3696.5–3701.0 cm^–1^, consistent with the range of values that have been reported for the air–water interface of liquid water.^63^ These results indicate that the structure of water and the electric field at the surface of nanodrops with diameters above a few tens of nm should be essentially the same as that at the bulk air–water interface. Thus, the ionization energies of these larger droplets should also be essentially the same as that of bulk water, which would preclude the occurrence of spontaneous ionization. Contact electrification of a neutral droplet with air could occur with some form of activation.^66^ This could be provided by the slip velocity in the aerodynamic acceleration that occurs in the atmospheric interface, although this effect is likely minor due to the short time droplets are accelerated. Considering these factors along with the experimental data, the predominant mechanism of ion production is ion-surface interactions between the neutral droplets and the steel capillary leading to contact electrification (ionization) of the droplets.

## Conclusions

Charge detection measurements show that highly charged water microdroplets are produced from neutral water microdroplets as they are introduced into a mass spectrometer through a typical atmospheric interface. Microdroplets with a few thousand to more than 200,000 positive charges were detected. There are two mechanisms for formation of these highly charged ions. The predominant mechanism is ion-surface interactions in the interface capillary that lead to the stripping of many electrons from the droplets to form highly positively charged droplets. A minor mechanism in which neutral droplets are broken up owing to rapidly reducing pressures and possibly shear forces from turbulent gas flow must also occur and leads to both positively and negatively charged droplets. The driving energy for these endothermic processes is the vacuum differential created by vacuum pumps, as well as collisional or thermal activation within the atmospheric interface.

These results show that many reactions observed using mass spectrometry that have been attributed to the high intrinsic electric field at the surface of uncharged water droplets have almost certainly occurred with highly charged droplets, consistent with these same reactions being observed in charged droplets formed by ESI or pneumatic nebulization. Microdroplets with kinetic energies up to hundreds of MeV are produced by aerodynamic acceleration in typical atmospheric sampling interfaces of mass spectrometers. Even a few MeV is sufficient to overcome the activation barriers of many chemical reactions, including oxidation of water microdroplets through the removal of thousands and even hundreds of thousands of electrons. Electrochemical oxidation that occurs in these droplet experiments is well-known to drive endothermic chemical reactions, and such reactions may also occur with analytes that are contained in these initially neutral or charged droplets that interact with the capillary (or other electrode surfaces).^47,67,68^

Zhang and co-workers^6^ note that the intrinsic electric field at the surface of neutral droplets can also accelerate the rates of chemical reactions. While this may be true for a number of reasons,^1,47^ this and most other studies that report reaction acceleration typically do not take into account droplet evaporation and reagent concentration that occurs in atmospheric sampling mass spectrometry experiments.^47,69^ Significant droplet evaporation occurs prior to gaseous ion formation and leads to formation of unsolvated gaseous ions that are multiply charged as demonstrated here. Reagents in the droplets become more concentrated as a result of this solvent loss. Evaporation occurs both in the metal capillary that is typically heated^70^ and inside the mass spectrometer itself.^31,32^ Rates of bimolecular reactions depend strongly on reagent concentration. For a bimolecular redox reaction for which no surface-effects are expected, rate acceleration factors between 10^2^ and 10^7^ were observed.^69^ These rates depended on the initial nanodroplet size and the initial starting concentration of the two reagents.^69^ These results show that reagent concentration occurs as solvent evaporates and droplets decrease in size and that the resulting rate acceleration is comparable to the range of rate acceleration that has often been attributed to the electric field at droplet surfaces. In essence, the likelihood of two different reagent molecules contained in a 1 μm diameter droplet (∼17.4 billion water molecules) finding each other and reacting is low, whereas this interaction is nearly certain when only a dozen water molecules remain with the two reagent molecules at late stages of droplet evaporation. While this description significantly oversimplifies the complex physical processes that occur with the highly charged droplets formed in these and many other experiments, including fission,^30–32^ charge emission,^32,71^ and molecular diffusion,^72^ this effect of reagent concentration needs to be considered in experiments where droplet evaporation occurs. Similarly, effects of activation either in initial droplet formation/ionization, such as electron emission from negatively charged droplets that are highly charged,^73^ or subsequent effects of activation that occur in ambient ionization interfaces as demonstrated here should also be taken into account before attributing unusual reaction chemistry to the intrinsic electric field at the surface of charged or uncharged water droplets. The phenomena that are described here must also occur in ESI experiments done in many thousands of laboratories worldwide to analyze a wide range of molecules and molecular assemblies.^74^ Although only a fraction of droplets interact with the interface capillary, the resulting droplet oxidation may provide insights into some otherwise unexplained chemistry that may occur. These results may also provide new insights into unusual reactions with water microdroplets that have been reported to occur spontaneously to produce abundant products, such as hydrogen peroxide,^67,75^ nitrogen fixation without a catalyst^76^ and abundant hydrogen gas generation from ammonia.^77^

## Methods

### Charge Detection with an Array Detector

Droplet experiments were done using a custom-built charge detection instrument (Figure 1a) that has an atmospheric sampling interface similar to those of commercial instruments. This interface consists of a 11.4 cm long (0.5 mm ID) stainless-steel capillary that has atmospheric pressure on one side and a pressure of ∼3 Torr on the other side. The capillary was 140 °C in ESI experiments and for one set of neutral droplet experiments (Figure S4). The capillary was 30 °C for all other experiments. The capillary exits into an ion funnel, and this region is followed by differentially pumped stages that include a quadrupole and a series of ion guides prior to the array detector. In these experiments, *all ion optics, including the ion funnel, quadrupole, and ion guides, were grounded so that all regions throughout the entire instrument were electric field-free.* The array detector consists of two sets of differentially amplified tubes (each tube is 10.16 mm long and has an inner diameter of 2.54 mm with a 1.53 mm spacing between tubes). There are 12 tubes per differential channel for a total of 24 tubes in the detector assembly that are arranged in an alternating pattern. The amplitude of signals induced by an ion (or charged droplet) as it passes through each tube is a direct measure of the particle charge. The induced signal was calibrated using a 2 pF test capacitor connected to an external signal equivalent to 60,000 charges. Current measurements on the stainless-steel interface capillary were made using a 485 autoranging picoammeter (Keithley Instruments, Solon, OH). A detailed description of data acquisition, data analysis, and other mass spectrometry experiments is provided in the Supporting Information.

### Water Droplet Generation

Three different methods were used to produce water droplets: electrospray ionization, a vibrating mesh nebulizer, and condensation using liquid N_2_. Both positively and negatively charged droplets were produced by ESI from an aqueous 200 μM NaCl solution (Figure 1b). Borosilicate capillaries (1.5 mm outer diameter, Sutter Instruments, Novato, CA) were pulled to an inner diameter of 2.4 μm with a Flaming Brown P-87 filament puller (Sutter Instruments, Novato, CA) and electrical contact to the solution was made with a platinum wire. Typical spray voltages were +1.7 kV and −2.5 kV for positive and negative droplet experiments, respectively. Bipolar droplets were generated using a mechanical vibrating mesh nebulizer (Meowyn, purchased from Amazon) that was positioned orthogonally roughly 10 cm away from the instrument inlet (Figure 1c). There were no electrical potentials applied to the solution in this nebulizer. Neutral water droplets were generated through condensation that occurred near an open dewar of liquid N_2_ (Figure 1d). No unexpected or unusual safety hazards were encountered, but proper precautions must be taken when working with high voltages and liquid N_2_, which is cold.

## Supplementary Material







## Notes

Video of cryogenic ionization with the charge detection instrument (MOV)
Video of cryogenic ionization with the Orbitrap instrument (MOV)
Description of software analysis, current measurements collected from the charge detection instrument capillary, droplet charging distribution for neutral droplets introduced via a heated capillary, photographs of relevant experimental apparatuses, charge detection mass spectrometer data for droplet and analytes, cryogenic ionization mass spectra with no liquid N_2_, cryogenic ionization mass spectra of laboratory air, and ESI mass spectra for comparison with cryogenic ionization data (PDF)

## References

[1] Wei Z., Li Y., Cooks R. G., Yan X. (2020). Accelerated Reaction Kinetics in
Microdroplets: Overview and Recent Developments. Annu. Rev. Phys. Chem..

[2] Rainer T., Eidelpes R., Tollinger M., Müller T. (2021). Microdroplet
Mass Spectrometry Enables Extremely Accelerated Pepsin Digestion of
Proteins. J. Am. Soc. Mass Spectrom..

[3] Müller T., Badu-Tawiah A., Cooks R. G. (2012). Accelerated Carbon-Carbon Bond-Forming
Reactions in Preparative Electrospray. Angew.
Chem., Int. Ed..

[4] Sahota N., Abusalim D. I., Wang M. L., Brown C. J., Zhang Z., El-Baba T. J., Cook S. P., Clemmer D. E. (2019). A Microdroplet-Accelerated
Biginelli Reaction: Mechanisms and Separation of Isomers Using IMS-MS. Chem. Sci..

[5] Holden D. T., Morato N. M., Cooks R. G. (2022). Aqueous Microdroplets Enable Abiotic
Synthesis and Chain Extension of Unique Peptide Isomers from Free
Amino Acids. Proc. Natl. Acad. Sci. U. S. A..

[6] Chen H., Li X., Li B., Chen Y., Ouyang H., Li Y., Zhang X. (2025). Microdroplet
Chemistry with Unactivated Droplets. J. Am.
Chem. Soc..

[7] Wang M., Gao X. F., Su R., He P., Cheng Y. Y., Li K., Mi D., Zhang X., Zhang X., Chen H., Cooks R. G. (2022). Abundant
Production of Reactive Water Radical Cations
under Ambient Conditions. CCS Chem..

[8] Qiu L., Cooks R. G. (2022). Simultaneous and
Spontaneous Oxidation and Reduction
in Microdroplets by the Water Radical Cation/Anion Pair. Angew. Chem., Int. Ed..

[9] Xing D., Meng Y., Yuan X., Jin S., Song X., Zare R. N., Zhang X. (2022). Capture of Hydroxyl Radicals by Hydronium
Cations in Water Microdroplets. Angew. Chem.,
Int. Ed..

[10] Lee J. K., Walker K. L., Han H. S., Kang J., Prinz F. B., Waymouth R. M., Nam H. G., Zare R. N. (2019). Spontaneous Generation
of Hydrogen Peroxide from Aqueous Microdroplets. Proc. Natl. Acad. Sci. U. S. A..

[11] Song Z., Zhu C., Gong K., Wang R., Zhang J., Zhao S., Li Z., Zhang X., Xie J. (2024). Deciphering the Microdroplet Acceleration
Factors of Aza-Michael Addition Reactions. J.
Am. Chem. Soc..

[12] Xing D., Gao X., Chen H., Zhang J., Edwards M. E., Liang C., Zhu C., Meng Y., Zare R. N., Xia Y., Zhang X. (2025). Challenges
in Detecting Hydroxyl Radicals Generated in Water Droplets with Mass
Spectrometry. Anal. Chem..

[13] Özdemir A., Lin J. L., Wang Y. S., Chen C. H. (2014). A Deeper Look into
Sonic Spray Ionization. RSC Adv..

[14] Xia Y., Xu J., Li J., Chen B., Dai Y., Zare R. N. (2024). Visualization
of the Charging of Water Droplets Sprayed into Air. J. Phys. Chem. A.

[15] Banerjee S., Zare R. N. (2015). Syntheses of Isoquinoline and Substituted
Quinolines
in Charged Microdroplets. Angew. Chem., Int.
Ed..

[16] Song X., Lyu L., Xu J., Xing D., Zhang X., Zare R. N. (2025). Clarifying
the Identity of the *m*/*z* 36 Ion in
Water Microdroplet Mass Spectra. J. Phys. Chem.
A.

[17] Zhang X., Duan M., Chingin K., Balabin R., Liu L., Zhang X., Chen H. (2025). Detection and Characterization of
Water Radical Cations by Mass Spectrometry and Electron Paramagnetic
Resonance Spectroscopy. Anal. Chem..

[18] Chen C. J., Williams E. R. (2025). A Source of the
Mysterious *m*/*z* 36 Ions Identified:
Implications for the Stability of
Water and Unusual Chemistry in Microdroplets. ACS Cent. Sci..

[19] Chen C. J., Williams E. (2024). Are Hydroxyl Radicals Spontaneously
Generated in Unactivated
Water Droplets?. Angew. Chem., Int. Ed..

[20] Chen C. J., Williams E. R. (2025). An Alternative Explanation
for Ions Put Forth as Evidence
for Abundant Hydroxyl Radicals Formed Due to the Intrinsic Electric
Field at the Surface of Water Droplets. Anal.
Chem..

[21] Schmidt F. M., Vaittinen O., Metsälä M., Lehto M., Forsblom C., Groop P. H., Halonen L. (2013). Ammonia in Breath and
Emitted from Skin. J. Breath Res..

[22] Valente E. L., Araujo L. C., Carvalho S. T., Stahlhofer M. (2018). Breath Ammonia
as a Bioindicator of Protein Nutrition in Heifers. Livest. Sci..

[23] Hibbard T., Killard A. J. (2011). Breath Ammonia Levels in a Normal
Human Population
Study as Determined by Photoacoustic Laser Spectroscopy. J. Breath Res..

[24] Smith D., Spanel P., Davies S. (1999). Trace Gases in Breath
of Healthy
Volunteers When Fasting and after a Protein-Calorie Meal: A Preliminary
Study. J. Appl. Physiol..

[25] Nose K., Minzuno T., Yamane N., Kondo T., Ohtani H., Araki S., Tsuda T. (2005). Identification
of Ammonia in Gas
Emanated from Human Skin and Its Correlation with That in Blood. Anal. Sci..

[26] Pagnotti V. S., Inutan E. D., Marshall D. D., McEwen C. N., Trimpin S. (2011). Inlet Ionization:
A New Highly Sensitive Approach for Liquid Chromatography/Mass Spectrometry
of Small and Large Molecules. Anal. Chem..

[27] Pagnotti V. S., Chubatyi N. D., McEwen C. N. (2011). Solvent
Assisted Inlet Ionization:
An Ultrasensitive New Liquid Introduction Ionization Method for Mass
Spectrometry. Anal. Chem..

[28] Pagnotti V. S., Chakrabarty S., McEwen C. N. (2013). Carbonation and Other Super Saturated
Gases as Solution Modifiers for Improved Sensitivity in Solvent Assisted
Ionization Inlet (SAII) and ESI. J. Am. Soc.
Mass Spectrom..

[29] Rayleigh L. (1882). XX. On the
Equilibrium of Liquid Conducting Masses Charged with Electricity. London, Edinburgh, Dublin Philos. Mag. J. Sci..

[30] Smith J. N., Flagan R. C., Beauchamp J. L. (2002). Droplet
Evaporation and Discharge
Dynamics in Electrospray Ionization. J. Phys.
Chem. A.

[31] Hanozin E., Harper C. C., McPartlan M. S., Williams E. R. (2023). Dynamics of Rayleigh
Fission Processes in ∼100 nm Charged Aqueous Nanodrops. ACS Cent. Sci..

[32] Avadhani V. S., Harper C. C., Miller Z. M., Williams E. R. (2025). Spontaneous
Fission
of Charged Water Nanodrops: Unveiling the Stochastic Nature of Fission
Pathways and Dynamics. J. Am. Chem. Soc..

[33] Gamero-Castãno M. (2007). Induction
Charge Detector with Multiple Sensing Stages. Rev. Sci. Instrum..

[34] Gamero-Castano M. (2009). Retarding
Potential and Induction Charge Detectors in Tandem for Measuring the
Charge and Mass of Nanodroplets. Rev. Sci. Instrum..

[35] Smith J. W., Siegel E. E., Maze J. T., Jarrold M. F. (2011). Image Charge Detection
Mass Spectrometry: Pushing the Envelope with Sensitivity and Accuracy. Anal. Chem..

[36] Doussineau T., Kerleroux M., Dagany X., Clavier C., Barbaire M., Maurelli J., Antoine R., Dugourd P. (2011). Charging Megadalton
Poly­(Ethylene Oxide)­s by Electrospray Ionization. A Charge Detection
Mass Spectrometry Study. Rapid Commun. Mass
Spectrom..

[37] Barney B.
L., Daly R. T., Austin D. E. (2013). A Multi-Stage Image Charge Detector
Made from Printed Circuit Boards. Rev. Sci.
Instrum..

[38] Harper C.
C., Jordan J. S., Papanu S., Williams E. R. (2024). Characterization
of Mass, Diameter, Density, and Surface Properties of Colloidal Nanoparticles
Enabled by Charge Detection Mass Spectrometry. ACS Nano.

[39] Elliott A. G., Merenbloom S. I., Chakrabarty S., Williams E. R. (2017). Single Particle
Analyzer of Mass: A Charge Detection Mass Spectrometer with a Multi-Detector
Electrostatic Ion Trap. Int. J. Mass Spectrom..

[40] Fuerstenau S. D., Benner W. H. (1995). Molecular Weight
Determination of Megadalton DNA Electrospray
Ions Using Charge Detection Time-of-flight Mass Spectrometry. Rapid Commun. Mass Spectrom..

[41] Williams L. R., Gonzalez L. A., Peck J., Trimborn D., McInnis J., Farrar M. R., Moore K. D., Jayne J. T., Robinson W. A., Lewis D. K., Onasch T. B., Canagaratna M. R., Trimborn A., Timko M. T., Magoon G., Deng R., Tang D., De La Rosa Blanco E., Prévôt A. S. H., Smith K. A., Worsnop D. R. (2013). Characterization
of an Aerodynamic
Lens for Transmitting Particles Greater than 1 Micrometer in Diameter
into the Aerodyne Aerosol Mass Spectrometer. Atmos. Meas. Technol..

[42] Bailey A. G. (1984). Electrostatic
Spraying of Liquids. Phys. Bull..

[43] Lin S., Xu L., Chi Wang A., Wang Z. L. (2020). Quantifying Electron-Transfer in
Liquid-Solid Contact Electrification and the Formation of Electric
Double-Layer. Nat. Commun..

[44] Sun M., Lu Q., Wang Z. L., Huang B. (2021). Understanding Contact Electrification
at Liquid–Solid Interfaces from Surface Electronic Structure. Nat. Commun..

[45] Harper C. C., Miller Z. M., McPartlan M. S., Jordan J. S., Pedder R. E., Williams E. R. (2023). Accurate Sizing
of Nanoparticles Using a High-Throughput
Charge Detection Mass Spectrometer without Energy Selection. ACS Nano.

[46] Morato N. M., Cooks R. G. (2023). Desorption Electrospray Ionization Mass Spectrometry:
20 Years. Acc. Chem. Res..

[47] Rovelli G., Jacobs M. I., Willis M. D., Rapf R. J., Prophet A. M., Wilson K. R. (2020). A Critical Analysis
of Electrospray Techniques for
the Determination of Accelerated Rates and Mechanisms of Chemical
Reactions in Droplets. Chem. Sci..

[48] Harper C. C., Avadhani V. S., Hanozin E., Miller Z. M., Williams E. R. (2023). Dynamic
Energy Measurements in Charge Detection Mass Spectrometry Eliminate
Adverse Effects of Ion-Ion Interactions. Anal.
Chem..

[49] Harrison J., Risby K. M., Miles B. E. A., Hilditch T. G., Walker J. S., Bzdek B. R. (2025). The Role of Aerosol Liquid Water in Droplet-Assisted
Ionization Mass Spectrometry. Anal. Chem..

[50] Walker J. S., Bzdek B. R. (2025). Rapid and Sensitive
Chemical Analysis of Individual
Picolitre Droplets by Mass Spectrometry. Anal.
Chem..

[51] Apsokardu M. J., Kerecman D. E., Johnston M. V. (2021). Ion Formation in Droplet-Assisted
Ionization. Rapid Commun. Mass Spectrom..

[52] Groenewold G. S., Sauter A. D. (2013). Rapid Analysis of
Single Droplets of Lanthanide-Ligand
Solutions by Electrospray Ionization Mass Spectrometry Using an Induction-Based
Fluidics Source. Anal. Chem..

[53] Ross R. L., Sauter A. D., Limbach P. A. (2015). Induction
Based Fluidics (IBF) for
Droplet-Based Mass Spectrometric Analysis of Oligonucleotides. J. Mass Spectrom..

[54] Zilch L. W., Maze J. T., Smith J. W., Ewing G. E., Jarrold M. F. (2008). Charge
Separation in the Aerodynamic Breakup of Micrometer-Sized Water Droplets. J. Phys. Chem. A.

[55] Maze J. T., Jones T. C., Jarrold M. F. (2006). Negative Droplets from Positive Electrospray. J. Phys. Chem. A.

[56] Pagnotti V. S., Chakrabarty S., Wang B., Trimpin S., McEwen C. N. (2014). Gas-Phase
Ions Produced by Freezing Water or Methanol for Analysis Using Mass
Spectrometry. Anal. Chem..

[57] Dykyj, J. ; Svoboda, J. ; Wilhoit, R. C. ; Frenkel, M. ; Hall, K. R. Vapor Pressure of Chemicals. In Landolt-Börnstein - Group IV Physical Chemistry; Hall, K. , Ed.; Springer-Verlag: Berlin Heidelberg, 2001; 10.1007/10688591_3.

[58] Perry C. F., Zhang P., Nunes F. B., Jordan I., Von Conta A., Wörner H. J. (2020). Ionization Energy of Liquid Water Revisited. J. Phys. Chem. Lett..

[59] Thürmer S., Malerz S., Trinter F., Hergenhahn U., Lee C., Neumark D. M., Meijer G., Winter B., Wilkinson I. (2021). Accurate Vertical
Ionization Energy and Work Function Determinations of Liquid Water
and Aqueous Solutions. Chem. Sci..

[60] Hao H., Leven I., Head-Gordon T. (2022). Can Electric
Fields Drive Chemistry
for an Aqueous Microdroplet?. Nat. Commun..

[61] Xiong H., Lee J. K., Zare R. N., Min W. (2020). Strong Electric Field
Observed at the Interface of Aqueous Microdroplets. J. Phys. Chem. Lett..

[62] Heindel J. P., Hao H., Lacour R. A., Head-Gordon T. (2022). Spontaneous
Formation of Hydrogen
Peroxide in Water Microdroplets. J. Phys. Chem.
Lett..

[63] Cooper R. J., O’Brien J. T., Chang T. M., Williams E. R. (2017). Structural and Electrostatic
Effects at the Surfaces of Size- and Charge-Selected Aqueous Nanodrops. Chem. Sci..

[64] O’Brien J. T., Williams E. R. (2012). Effects of Ions on Hydrogen-Bonding Water Networks
in Large Aqueous Nanodrops. J. Am. Chem. Soc..

[65] Prell J. S., O’Brien J. T., Williams E. R. (2011). Structural and Electric Field Effects
of Ions in Aqueous Nanodrops. J. Am. Chem. Soc..

[66] Wang F., Yang P., Tao X., Shi Y., Li S., Liu Z., Chen X., Wang Z. L. (2021). Study of
Contact Electrification
at Liquid-Gas Interface. ACS Nano.

[67] Eatoo M. A., Mishra H. (2024). Busting the Myth of
Spontaneous Formation of H_2_O_2_ at the Air–Water
Interface: Contributions
of the Liquid–Solid Interface and Dissolved Oxygen Exposed. Chem. Sci..

[68] Eatoo M. A., Wehbe N., Kharbatia N., Guo X., Mishra H. (2025). Why Do Some
Metal Ions Spontaneously Form Nanoparticles in Water Microdroplets?
Disentangling the Contributions of the Air-Water Interface and Bulk
Redox Chemistry. Chem. Sci..

[69] Chen C. J., Williams E. R. (2023). The Role of Analyte
Concentration in Accelerated Reaction
Rates in Evaporating Droplets. Chem. Sci..

[70] Xia Z., Williams E. R. (2019). Effect of Droplet
Lifetime on Where Ions Are Formed
in Electrospray Ionization. Analyst.

[71] Harper C. C., Brauer D. D., Francis M. B., Williams E. R. (2021). Direct Observation
of Ion Emission from Charged Aqueous Nanodrops: Effects on Gaseous
Macromolecular Charging. Chem. Sci..

[72] Wilson K. R., Prophet A. M., Rovelli G., Willis M. D., Rapf R. J., Jacobs M. I. (2020). A Kinetic Description
of How Interfaces Accelerate
Reactions in Micro-Compartments. Chem. Sci..

[73] Chen C. J., Avadhani V. S., Williams E. R. (2025). Electronic
Excitation and High-Energy
Reactions Originate From Anionic Microdroplets Formed by Electrospray
or Pneumatic Nebulization. Angew. Chem., Int.
Ed..

[74] Prabhu G. R. D., Williams E. R., Wilm M., Urban P. L. (2023). Mass Spectrometry
Using Electrospray Ionization. Nat. Rev. Methods
Primers.

[75] Chen B., Xia Y., He R., Sang H., Zhang W., Li J., Chen L., Wang P., Guo S., Yin Y., Hu L., Song M., Liang Y., Wang Y., Jiang G., Zare R. N. (2022). Water – Solid Contact Electrification Causes
Hydrogen Peroxide Production from Hydroxyl Radical Recombination in
Sprayed Microdroplets. Proc. Natl. Acad. Sci.
U. S. A..

[76] Wang Y., Luo J., Fang Y. G., Nan Z. A., Cui X., Chen T., Zeng X., Wang X., Song X., Zhao J., Li W., Zeng C., Chen D., Zhu C., Wei Z., Tian Z. Q., Fan F. R. (2025). Catalyst-Free Nitrogen Fixation by
Microdroplets through a Radical-Mediated Disproportionation Mechanism
under Ambient Conditions. J. Am. Chem. Soc..

[77] Li K., Zhang B., Xia D., Ye Z., Pan Y., Francisco J. S., Mi Z. (2025). Room-Temperature Catalyst-Free
Ammonia
Decomposition for Hydrogen Production on Water Microdroplets. J. Am. Chem. Soc..

