# Supplementary material for: Atmospheric Sampling Mass Spectrometers Activate and Ionize Neutral Water Microdroplets to MeV Energies and up to 200,000+ Charges: Implications for Water Stability and Unusual Chemistry in Microdroplets
Source: ACS Cent Sci. 2025 Oct 31;11(12):2410–20. doi: 10.1021/acscentsci.5c01518 (PMC12746156; doi:10.1021/acscentsci.5c01518)
Supplement: Supplementary file 3 [file oc5c01518_si_003.pdf]

Supporting Information for

Atmospheric Sampling Mass Spectrometers Activate and Ionize Neutral Water Microdroplets to MeV Energies and up to 200,000+ Charges: Implications for Water Stability and Unusual Chemistry in Microdroplets

Matthew S. McPartlan,[1] Casey J. Chen,[1] Conner C. Harper,[1] Zachary M. Miller,[1] Julian Robles,[1] Veena S. Avadhani,[1] Randall E. Pedder,[2] Luke J. Metzler,[2] and Evan R. Williams[1]*

1. *Department of Chemistry, University of California, Berkeley, California 94720-1460, United States*
2. *Ardara Technologies LP, Ardara, Pennsylvania 15615, United States*

*To whom correspondence should be addressed

Email: erw@berkeley.edu

Supporting Information

Signal Processing and Data Analysis

Signal Processing. An ion passing through the linear charge detector array induces an image charge signal on each detector tube. There are 24 detector tubes in total, wired so that every other tube is connected together to form two channels. Detector tubes 1, 3, 5, etc. constitute channel **A** whereas detector tubes 2, 4, 6, etc. make up channel **B**. Preamplifiers in the vacuum chamber are used to amplify the output of each signal. These outputs are differentially amplified so that the final signal is channel **A**-**B**. This configuration was chosen so that a positively charged ion passing through the detector array induces a positive voltage signal in the first tube, and a negatively charged ion produces an initially negative signal. The signal is passed through an analog band-pass filter with a V/V gain of 10x (passband ~1.7 kHz – 300 kHz) and is digitized using a 12-bit ATS9120 digitizer card (Alazar Tech, Pointe-Claire, QC) at 2 MHz. The digitizing range of 0.4 V corresponds to a maximum of 200,000 *e* that can be measured before the signal saturates the digitizer. Ions where charge saturation occurs are assigned a minimum charge of 200,000. The detector signal is filtered a second time using a digital 4th order Butterworth high-pass filter with a cutoff frequency of 1,500 Hz to remove low frequency noise that affects the signal baseline. The filtered signal is then smoothed using a 4th order Savitzky-Golay filter with a window length of 50 μs to reduce noise while preserving the shape of the signal.

Verification of Neutral Droplet Formation. To verify that droplets formed by condensation with liquid N_2 are not initially charged, a vertical 2" diameter PVC tube with a 90º bend at the bottom was constructed to serve as a droplet guide to funnel droplets towards the instrument inlet. A copper mesh (1 mm mesh size) was positioned across the inlet to this pipe such that all droplets must pass through this mesh to reach the mass spectrometer inlet. A picoammeter

(Keithley Instruments, Solon, OH) was connected to the inlet capillary and droplets formed via cryogenic condensation were introduced to the entrance of the PVC tube. Potentials of -5 kV, 0 V, and +5 kV were applied to the copper mesh and the current generated on the inlet capillary was recorded. Consecutive inlet capillary current measurements were recorded over the course of several seconds. These data are shown in Figure S2. None of these potentials had a noticeable effect on either the current measured on the capillary nor the rate at which ions passed through the array detector. The high variability in the current that was measured is due to effects of the movement of laboratory air that could deflect the droplets from the instrument inlet. These results show that condensed microdroplets are initially neutral prior to entering the instrument.

Event Identification. The large number of charged droplets (over 100,000 charged droplets analyzed) necessitated a way to automatically determine the charge and the velocity of each ion. An ion that passes through all 24 tubes produces a square wave signal with 12 complete oscillation cycles. Less ideal ion signals can occur due to ion-ion interactions and overlapping signals when a large number of ions pass through the array at the same time. Ion collisions with the detector tube walls can also occur because there are no ion optics used to focus the ions that are produced through the detector array. Ion signals were identified by finding regions in the time domain data where the smoothed signal amplitude exceeded threshold values, set at ±3.0x the root-mean-square (RMS) noise of the signal. The RMS baseline noise is calculated by dividing each one-second acquisition signal into consecutive 50 ms segments and using the lowest value. This effectively creates a threshold of ~3,000 charges. This threshold was used in order to avoid lower amplitude signals that increased the error rate in assigning the charge and polarity of each droplet. For the analysis used in this work, events with between 10 and 25 associated threshold crossings were considered potentially valid ion signals. Threshold crossings were identified in

both the positive and negative direction. Valid crossing pairs were defined as consecutive crossings of opposite polarity with a separation between 25 μs and 200 μs. These crossing pairs were then grouped to form a complete ion signal. Ion signals that match these criteria were then checked for baseline stability prior to the ion entering the first tube. It is important that the detector signal returns to baseline in between ion signals when determining the direction of the first pulse. This was accomplished by checking for additional crossings of a lower set of thresholds (±2.0x RMS noise) in the 250 μs preceding the event. Events were rejected if more than two such threshold crossings were detected, and remaining events were saved for subsequent analysis to determine ion velocity and charge.

Effects of Temperature on Capillary Interactions. To determine if water condensate inside the metal capillary affected the results for data measured with a metal capillary at 30 °C, these experiments were repeated by introducing neutral droplets produced via cryogenic condensation with the capillary at 140 °C. Results from automated analysis of ion signals obtained from these data acquired at the higher temperature are shown in Figure S4 and resemble those obtained at 30 °C (Figure 3 in the main text). The greater population of negative droplets shown in Figure S4 is consistent with higher rates of mechanical breakup at increased temperatures.

Ion Velocity Determination. Ion velocity was determined from the time required for each particle to pass through a set of two detector tubes, corresponding to a single pair of threshold crossings in the time domain signal. The duration of each cycle was averaged, providing up to 12 separate measurements if the particle transited all 24 tubes. The distance a particle must travel within the detector array to complete a full cycle is 0.023 m. Combining this distance with the average cycle duration and number of cycles provides a direct measurement of ion velocity.

Ion Charge Determination. The charge of each ion that was identified was determined from the average peak-to-peak amplitude of the signal measured for each cycle. This amplitude was converted to elementary charges using a conversion factor determined through calibration of the instrument with an external signal equivalent to 60,000 *e* passed through a 2 pF capacitor.

Cryogenic Ionization Mass Spectrometry. Mass spectrometry experiments were performed using a Velos Pro dual pressure linear ion trap of an Orbitrap Elite instrument (Thermo Fisher Scientific, San Jose, CA) with a capillary temperature of either 40 °C or 100 °C. A heated metal plate containing a solid sample of 1,12-dodecanediamine or a Kimwipe with 10 μL glacial acetic acid was placed ~2 cm below the capillary inlet (Figure S6). Condensate droplets were formed at the top of a small dewar containing liquid N_2. All spectra were averaged over 1 min unless otherwise specified. For nanoelectrospray MS experiments, borosilicate capillaries with an inner diameter of 1.7 μm were used. A solution of 10 μM 1,12-dodecanediamine in methanol was introduced into the emitter. The emitter was placed approximately 3 mm from the capillary inlet, and an electrospray potential of 0.5 kV was applied to a platinum wire in contact with the solution. 1,12-dodecanediamine and glacial acetic acid were obtained from Sigma-Aldrich (St. Louis, MO). All chemicals were used without additional purification.

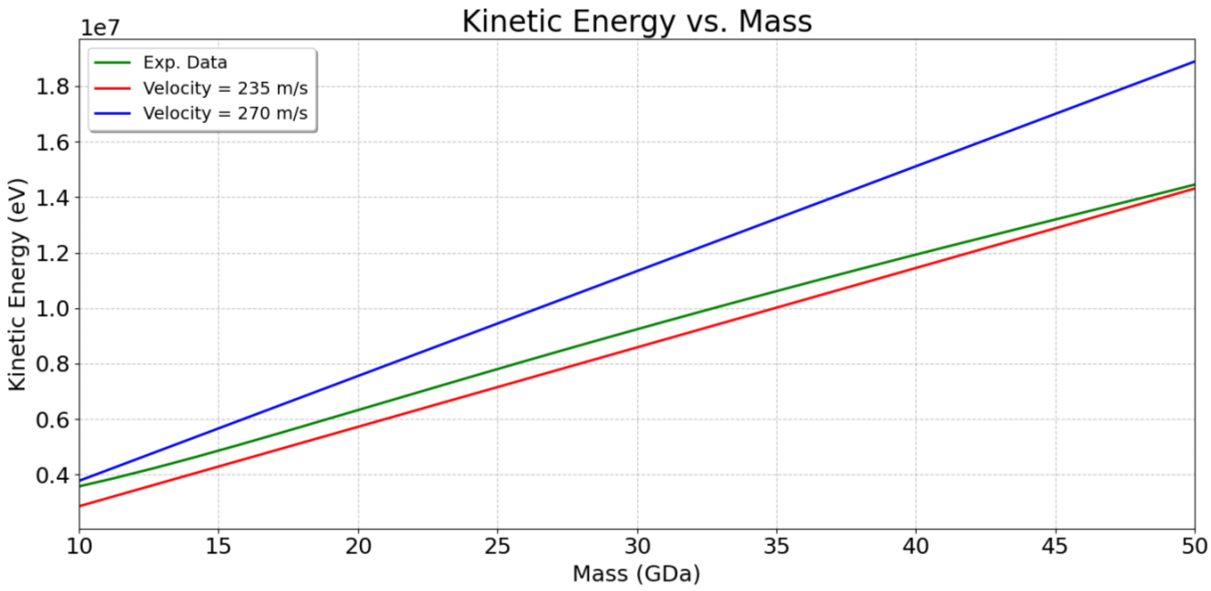

Figure S1. An expanded view of the average kinetic energy of charged microdroplets as a function of mass (green line) determined from the average velocity and estimated mass of the droplets shown in Figure 4b (purple line). Red and blue lines show the calculated kinetic energy as a function of mass for particles moving at a velocity of 235 m/s (red) and 270 m/s (blue). The average kinetic energy as a function of mass below 50 MDa (green line) has curvature, consistent with the measured decrease in the average velocities over this mass range (Figure 4a).

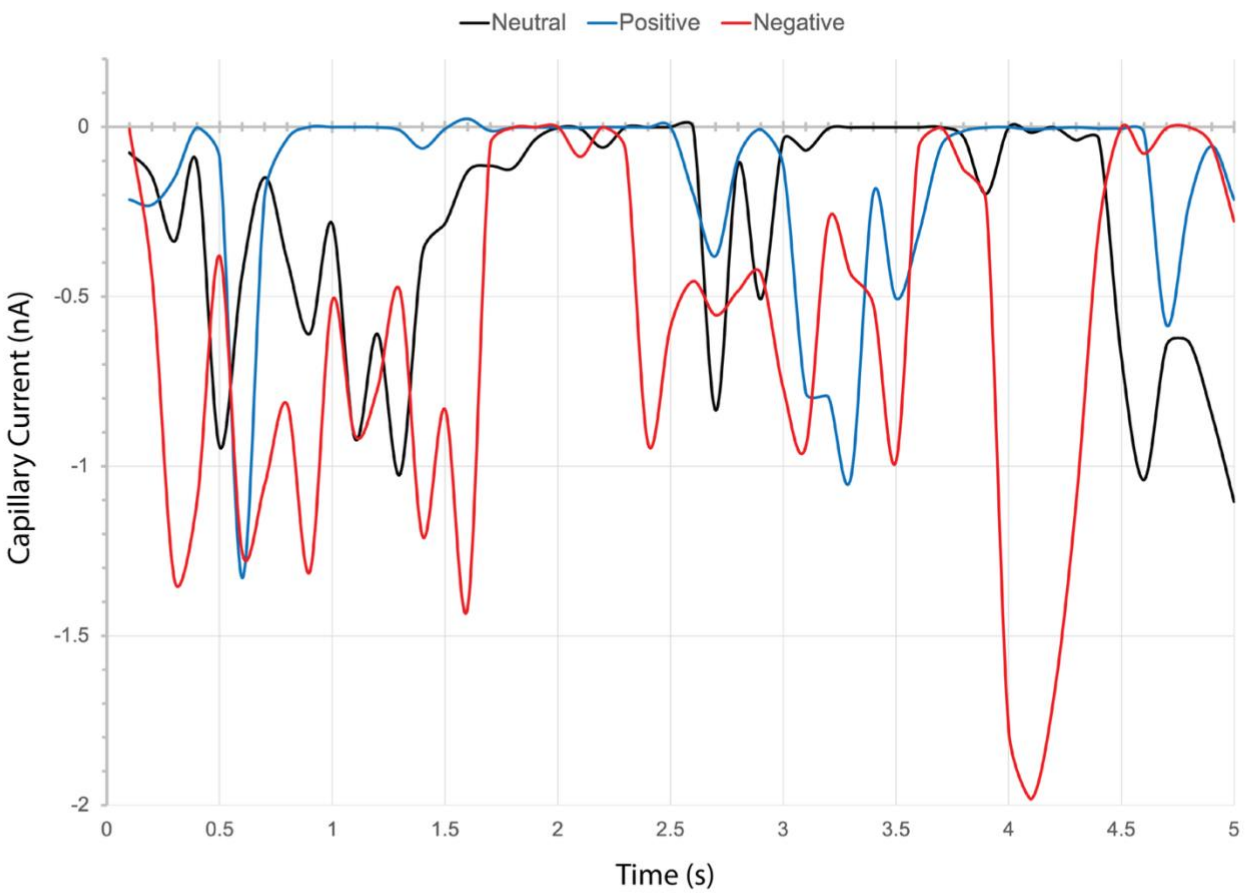

Figure S2. Inlet capillary current as a function of time with a copper wire mesh potential of 0 V, +5 kV, and -5 kV. Droplets must pass through the copper wire mesh in order to make it into the charge detection instrument. In all cases, a negative net capillary current was measured. These currents were -0.27 ± 0.34 nA, -0.15 ± 0.28 nA, and -0.58 ± 0.53 nA for the neutral, positive, and negative potentials, respectively. The instability in the ion current is attributed to observable fluctuations in air currents and condensate volume that reached the inlet during these experiments.

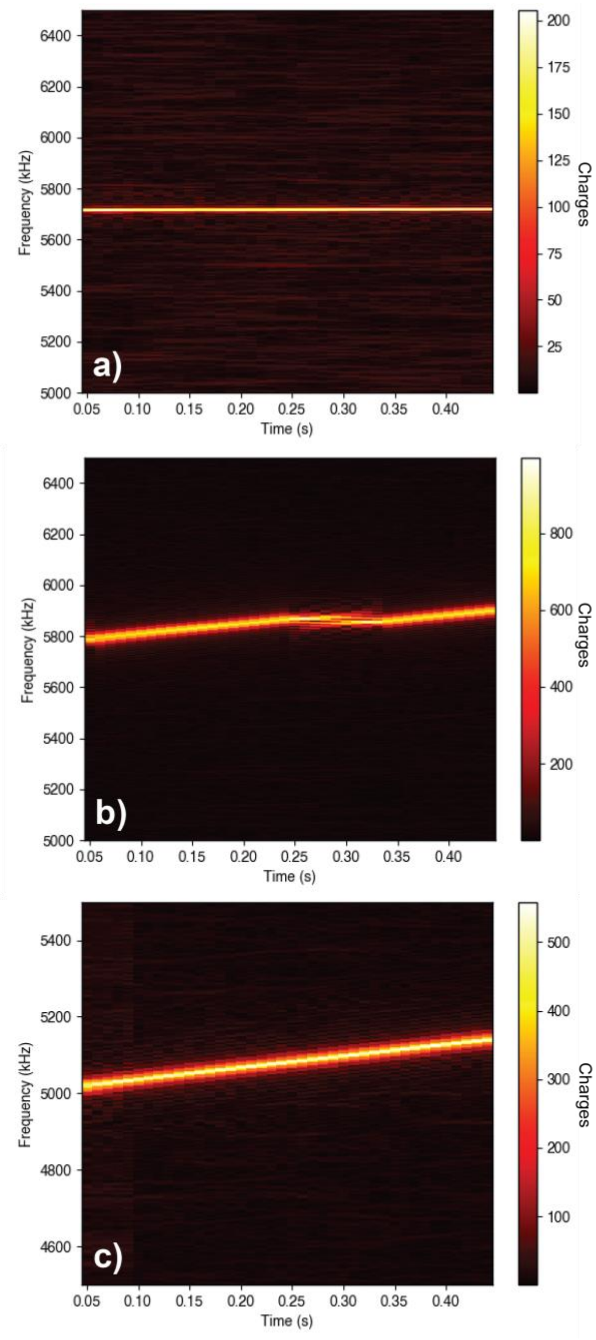

Figure S3. Results from charge detection mass spectrometry measurements performed in a linear electrostatic ion trap instrument[1] showing ion signals as a function of trap time. These signals correspond to the fundamental frequency of ion motion within the trap. In these experiments, positive electrospray was used to measure the mass distribution of a sample of 50 nm diameter polystyrene nanospheres. These measurements were followed by positive electrospray of pure

water without cleaning the mass spectrometer interface capillary. With ESI of just pure water, ions with both nearly constant frequencies and rapidly changing frequencies were observed. Examples of both types of ion signals that have similar initial oscillation frequencies are shown in a) and b), respectively. The mass of the ion in a) is 61.6 MDa, consistent with a spherical particle diameter of ~58 nm. The nearly constant frequency of this ion signal shows that this particle is not hydrated to a measurable extent and thus corresponds to a polystyrene nanosphere from the previous sample that was picked up from the surface of the capillary by a charged water droplet. The slope of the ion frequency in b) increases rapidly with time due to a loss of mass from evaporation of water[2,3] indicating that this must be a charged water droplet. This droplet has an initial mass of 357.2 MDa and a final mass of 346.7 MDa, indicating that ~10.5 MDa was lost from this droplet over a 0.5 s trap time. In addition to mass loss, the charged nanodroplet undergoes a fission process that occurs at 30 ms,[2,3] confirming its identity as a charged aqueous nanodrop. A trapped water droplet that did not undergo fission (or charge emission) is shown in c). This ~96 nm diameter water nanodroplet had an initial and final mass of 278.0 MDa and 270.6 MDa, respectively, corresponding to the loss of ~7.4 MDa, or roughly 411,000 H_2O molecules, over a 0.5 s trap time. The observation of high mass ion signals that do not undergo a significant frequency change with time confirms that charged water droplets interact with the surface of the capillary and can pick up large analytes from these interactions.

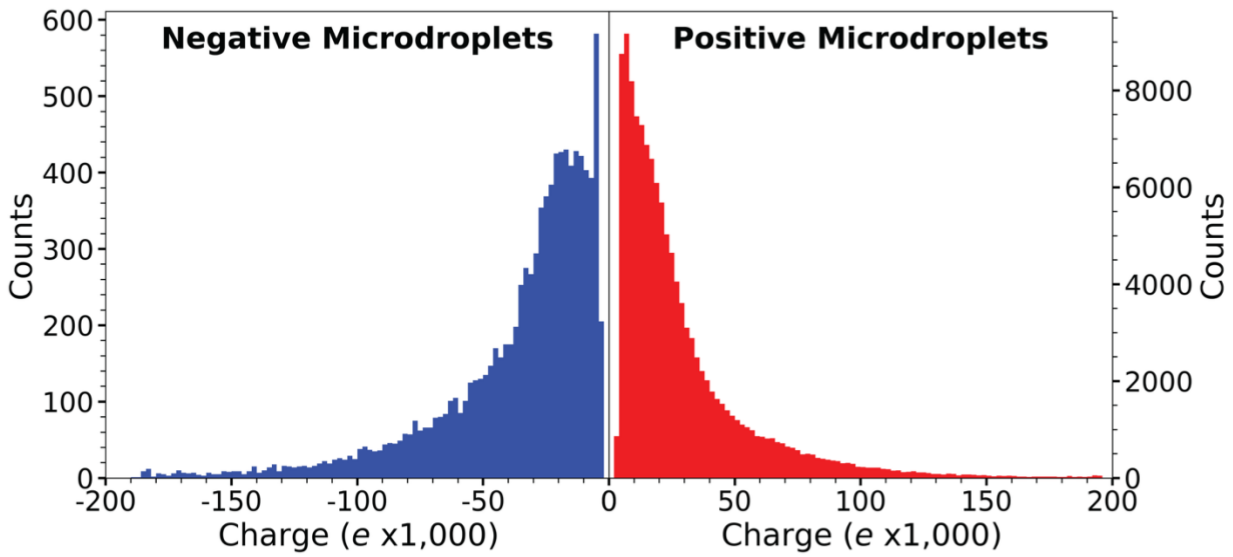

Figure S4. The population of droplets as a function of individual droplet charge for initially neutral droplets generated by condensation with liquid nitrogen that were introduced into the charge detection instrument with a capillary temperature of 140 °C. Negatively (9,766 ions) and positively (124,024 ions) charged droplet data are shown in blue and red, respectively. All droplet data was automatically identified by the analysis software and were not manually reviewed. As was the case for the data obtained at 30 °C, the error rate for the negative ions will be higher (see text). However, these data show that significantly more positive droplets are formed at the higher temperature indicating that the droplets interact with the steel surface and not condensed water.

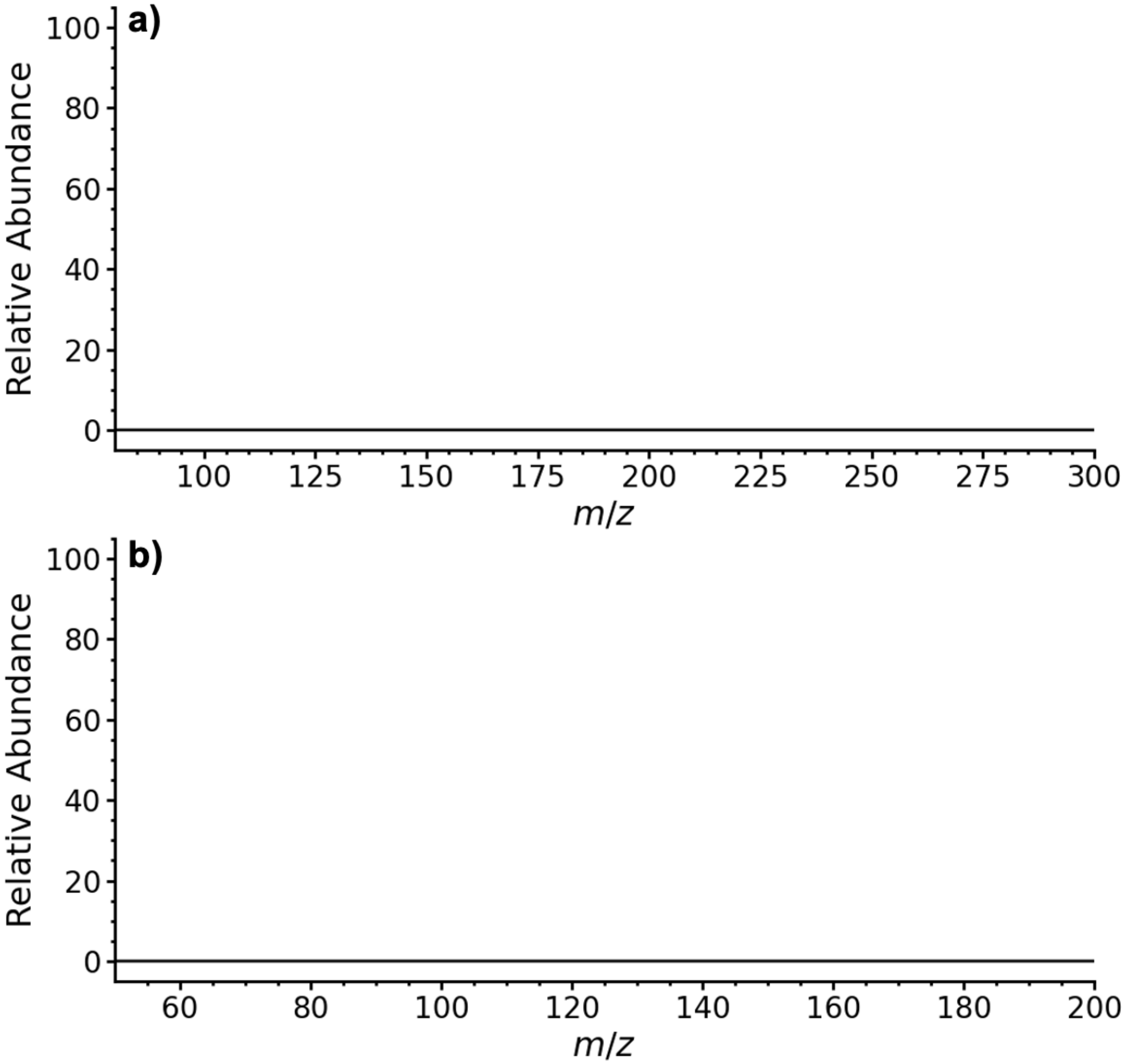

Figure S5. Mass spectra of vapor from a) 1,12-dodecanediamine and b) acetic acid with the sample present as shown in Figure S6 but with no liquid N_2 present to form condensate. The signals for these spectra were averaged for 1.4 and 1.0 min, respectively. No ion signal was detected in the absence of liquid N_2, indicating that these compounds do not spontaneously ionize and that condensate formed by liquid N_2 is responsible for the signals shown in Figure 7.

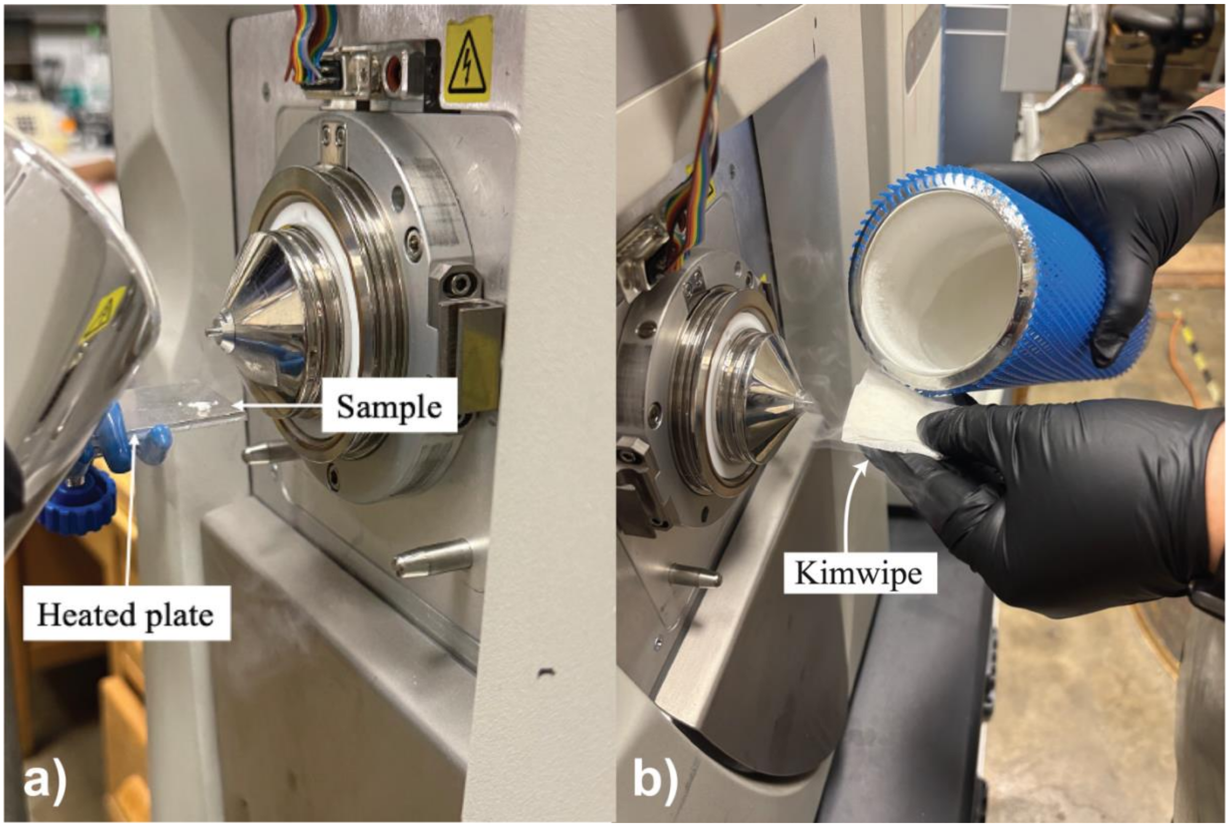

Figure S6. Image of droplet and vapor generation in front of the mass spectrometer inlet (measurements made in a Velos Pro dual pressure linear ion trap of an Orbitrap Elite instrument) with either a) a heated plate or b) a Kimwipe. The back end of the plate was heated with a heat gun and the sample was placed approximately 2 cm below the mass spectrometer inlet. No heating was used with the Kimwipe, which was also held approximately 2 cm below the mass spectrometer inlet.

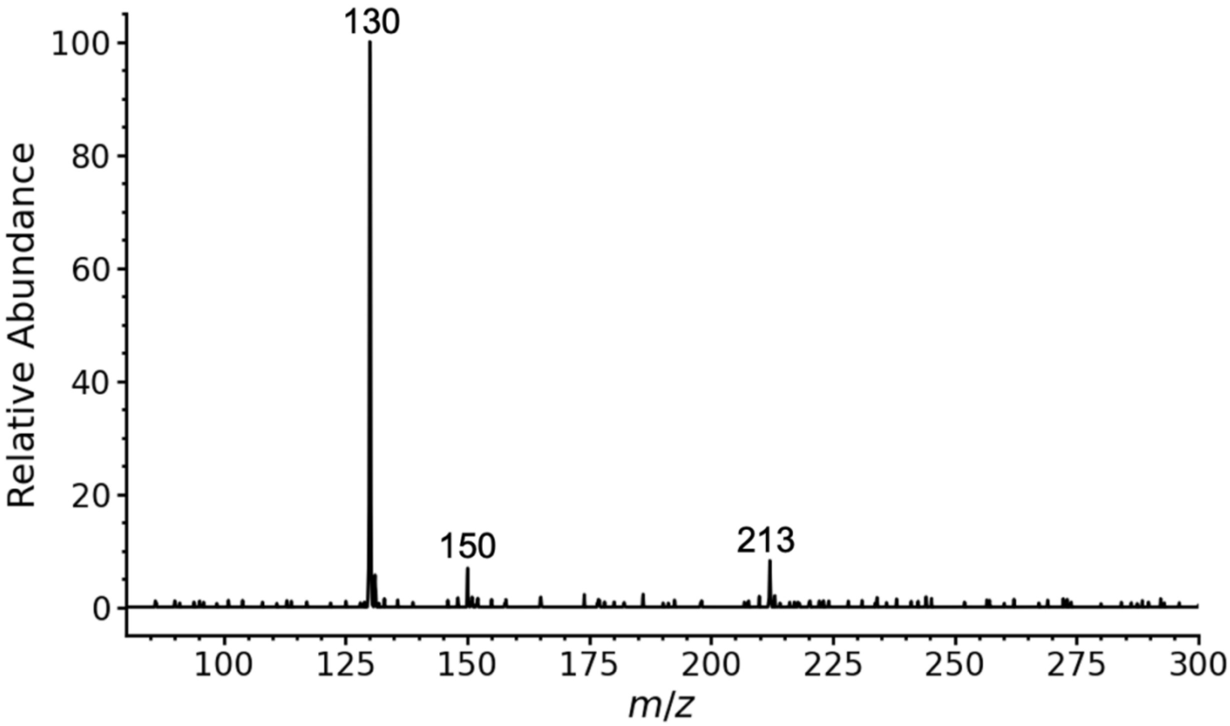

Figure S7. Mass spectrum generated by forming condensate from liquid N_2 with no sample intentionally placed near the instrument. An abundant ion at *m*/*z* 130 is observed indicating that this compound is likely a contaminant in the laboratory air. The ion mass indicates that this compound, if protonated, contains one or more nitrogen atoms, consistent with the compound having a high gas-phase basicity.

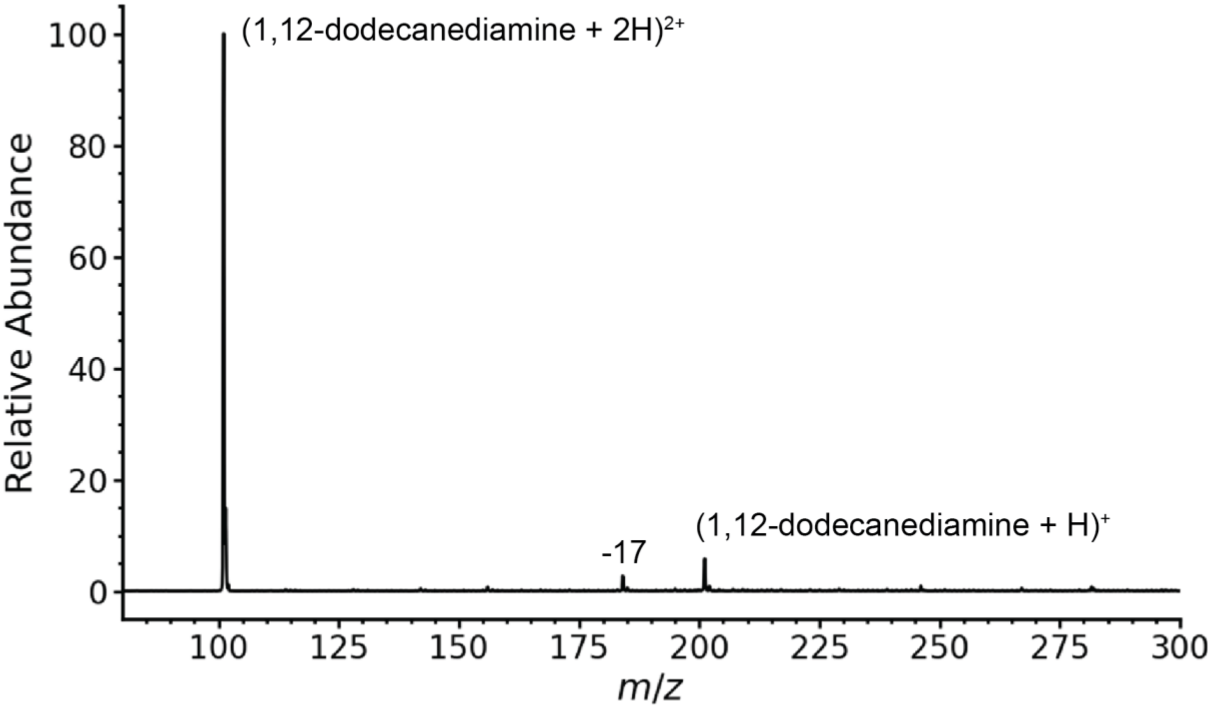

Figure S8. Positive electrospray ionization mass spectrum of 10 μM 1,12-dodecanediamine in methanol. The peak at *m*/*z* 184 is a fragmentation product corresponding to loss of NH_3. Ion signal at *m*/*z* 130 that is present in the liquid N_2 condensate experiments was not present in this spectrum indicating that this compound is not an impurity in this sample.

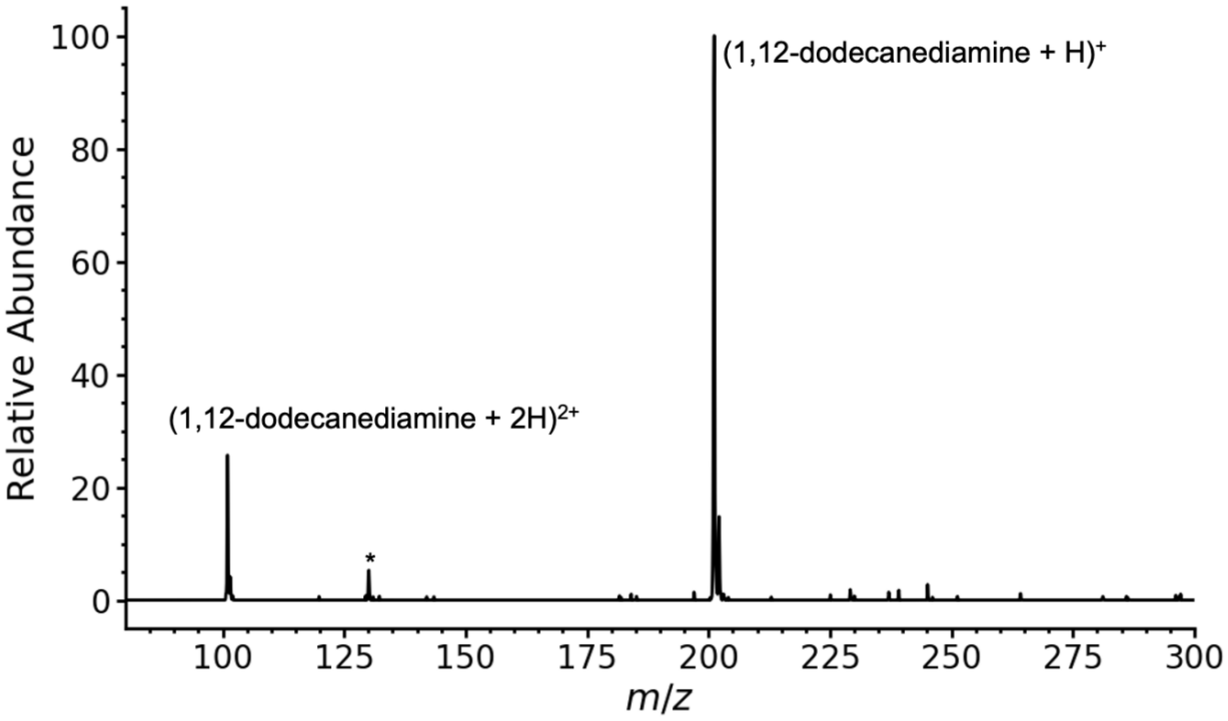

Figure S9: Cryogenic ionization mass spectrum of 1,12-dodecanediamine vapor and droplets formed from the condensate of liquid N_2 with a capillary temperature of 100 °C. The signal abundance is 2.7x that of the data measured at 40 °C.

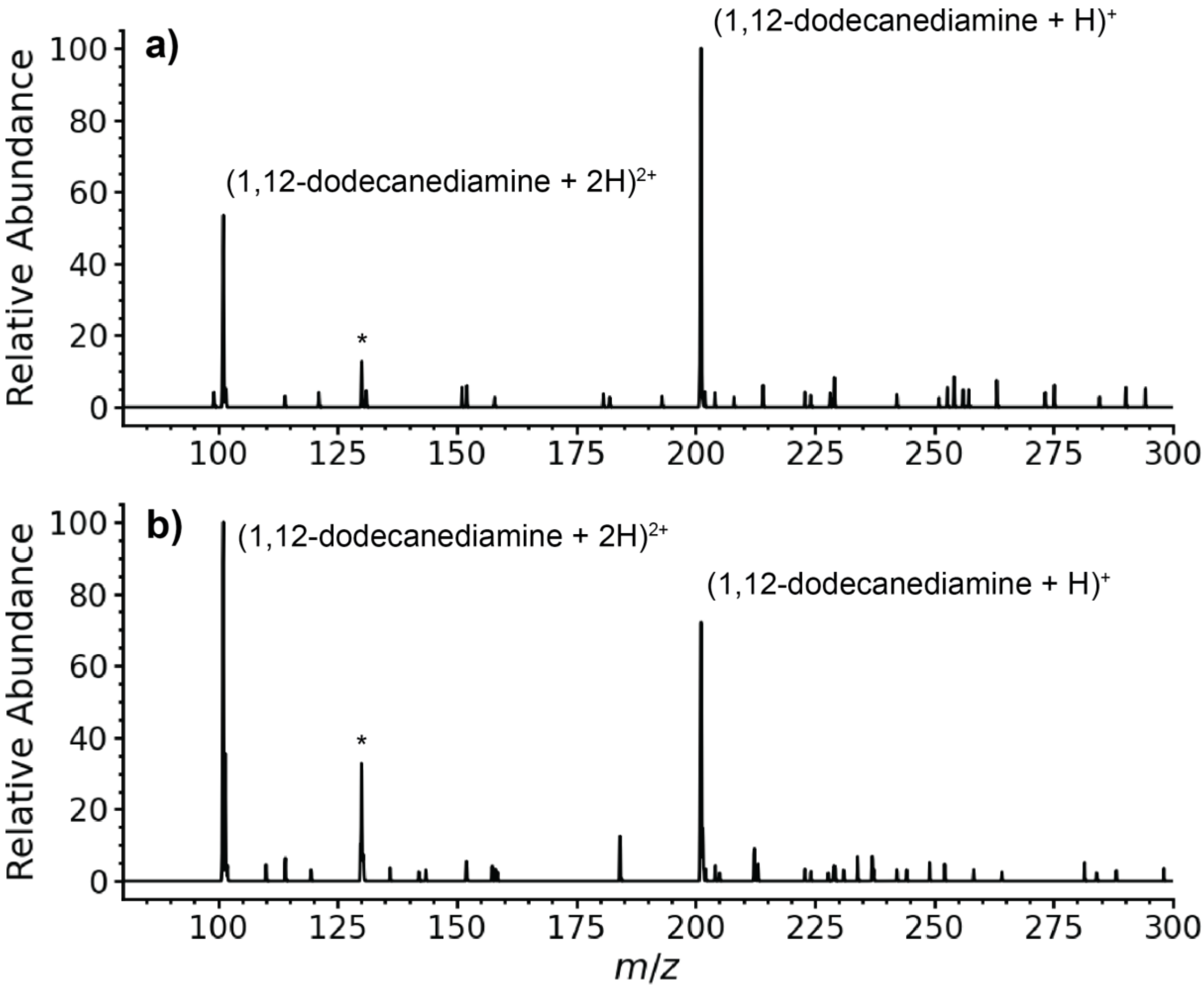

Figure S10: Cryogenic ionization mass spectra of 1,12-dodecanediamine vapor formed with an unheated solid sample positioned ~2 cm below the entrance to the mass spectrometer and exposed to condensate droplets formed with liquid N_2 with the atmospheric region in front of the mass spectrometer a) exposed to ambient laboratory light, and b) inside an enclosure that blocked light from the entire sample introduction region of the mass spectrometer. The normalization level (NL) values for the spectra are 0.82 and 0.87, respectively, indicating that exposure to light had no effect on the ion abundance. The signal abundance is lower than that in Figure 5a because the sample was not heated leading to a lower concentration of sample vapor.

References

(1) Harper, C. C.; Miller, Z. M.; McPartlan, M. S.; Jordan, J. S.; Pedder, R. E.; Williams, E. R. Accurate Sizing of Nanoparticles Using a High-Throughput Charge Detection Mass Spectrometer without Energy Selection. *ACS Nano* **2023**, *17*, 7765–7774.

(2) Avadhani, V. S.; Harper, C. C.; Miller, Z. M.; Williams, E. R. Spontaneous Fission of Charged Water Nanodrops: Unveiling the Stochastic Nature of Fission Pathways and Dynamics. *J. Am. Chem. Soc.* **2025**, *147*, 18853–18863.

(3) Hanozin, E.; Harper, C. C.; McPartlan, M. S.; Williams, E. R. Dynamics of Rayleigh Fission Processes in ~100 nm Charged Aqueous Nanodrops. *ACS Cent. Sci.* **2023**, *9*, 1611–1622.
